# Evolutionary trait‐based approaches for predicting future global impacts of plant pathogens in the genus *Phytophthora*

**DOI:** 10.1111/1365-2664.13820

**Published:** 2020-12-23

**Authors:** Louise J. Barwell, Ana Perez‐Sierra, Beatrice Henricot, Anna Harris, Treena I. Burgess, Giles Hardy, Peter Scott, Nari Williams, David E. L. Cooke, Sarah Green, Daniel S. Chapman, Bethan V. Purse

**Affiliations:** ^1^ UK Centre for Ecology and Hydrology Wallingford UK; ^2^ Forest Research Alice Holt Lodge Farnham UK; ^3^ Forest Research Northern Research Station Roslin UK; ^4^ Phytophthora Science and Management Centre for Climate Impacted Terrestrial Ecosystems Harry Butler Institute Murdoch University Murdoch Australia; ^5^ Plant & Food Research Te Puke New Zealand; ^6^ Plant and Food Research Havelock North New Zealand; ^7^ The James Hutton Institute Dundee UK; ^8^ Biological and Environmental Sciences University of Stirling Stirling UK

**Keywords:** biosecurity, global transport, horizon scanning, host range, invasiveness, pathogen, plant health, traits

## Abstract

1. Plant pathogens are introduced to new geographical regions ever more frequently as global connectivity increases. Predicting the threat they pose to plant health can be difficult without in‐depth knowledge of behaviour, distribution and spread. Here, we evaluate the potential for using biological traits and phylogeny to predict global threats from emerging pathogens.
2. We use a species‐level trait database and phylogeny for 179 *Phytophthora* species: oomycete pathogens impacting natural, agricultural, horticultural and forestry settings. We compile host and distribution reports for *Phytophthora* species across 178 countries and evaluate the power of traits, phylogeny and time since description (reflecting species‐level knowledge) to explain and predict their international transport, maximum latitude and host breadth using Bayesian phylogenetic generalised linear mixed models.
3. In the best‐performing models, traits, phylogeny and time since description together explained up to 90%, 97% and 87% of variance in number of countries reached, latitudinal limits and host range, respectively. Traits and phylogeny together explained up to 26%, 41% and 34% of variance in the number of countries reached, maximum latitude and host plant families affected, respectively, but time since description had the strongest effect.
4. Root‐attacking species were reported in more countries, and on more host plant families than foliar‐attacking species. Host generalist pathogens had thicker‐walled resting structures (stress‐tolerant oospores) and faster growth rates at their optima. Cold‐tolerant species are reported in more countries and at higher latitudes, though more accurate interspecific empirical data are needed to confirm this finding.
5. *Policy implications*. We evaluate the potential of an evolutionary trait‐based framework to support horizon‐scanning approaches for identifying pathogens with greater potential for global‐scale impacts. Potential future threats from *Phytophthora* include *Phytophthora x heterohybrida*, *P. lactucae*, *P. glovera*, *P. x incrassata*, *P. amnicola* and *P. aquimorbida*, which are recently described, possibly under‐reported species, with similar traits and/or phylogenetic proximity to other high‐impact species. Priority traits to measure for emerging species may be thermal minima, oospore wall index and growth rate at optimum temperature. Trait‐based horizon‐scanning approaches would benefit from the development of international and cross‐sectoral collaborations to deliver centralised databases incorporating pathogen distributions, traits and phylogeny.

## Graphical abstract

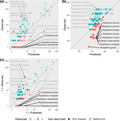

## 1 INTRODUCTION

Unintentional introductions of non‐native plant pathogens are a major cause of emerging diseases of plants and threaten agricultural, horticultural, forestry and natural ecosystems (Bebber et al., 2014). The top 100 of the World's Worst Invasive Alien Species includes the causal agents of Dutch elm disease *Ophiostoma novo‐ulmi*, chestnut blight *Cryphonectria parasitica* and an oomycete pathogen *Phytophthora cinnamomi* known to cause disease in at least 900 tree species (Lowe et al., 2000). In the United States, invasive plant pathogens cost an estimated $21 billion in crop losses and $2.1 billion in lost forest products each year (Pimentel et al., 2005). Developing tools for the early identification of future threats from pathogens with greater potential for global transport, establishment at higher latitudes and broad host ranges can help to inform plant health horizon‐scanning activities and improve preparedness.

Horizon scanning is increasingly recognised as central to invasive species management (Shine et al., 2010) particularly as emphasis has shifted towards preventative, rather than reactive management. At the global scale, ranked lists of invasive species including the IUCN ‘100 of the World's Worst Invasive Alien Species’ (Lowe et al., 2000) and the DAISIE (Delivering Alien Species Inventories for Europe) ‘100 of the Worst’ were developed to raise awareness and support biosecurity policy. European Union Regulation (No 1143/2014) on the prevention and management of the introduction and spread of invasive alien species (IAS) led to a curated list of 66 species of Union Concern (Roy et al., 2015, 2019). The methodology was adapted from previous approaches used to rank IAS threats to Great Britain (Roy et al., 2014, 2017) and has since been used for regional prioritisation of invasive species (Gallardo et al., 2016). A challenge for horizon scanning is the scarcity of data for evaluating future threats and the process usually involves a rigorous process of expert elicitation and consensus building to reduce bias and uncertainty (Roy et al., 2019). Opportunities to integrate empirical, quantitative approaches into horizon‐scanning exercises have yet to be fully explored.

Phylogenetic relatedness has proved a useful predictor of host susceptibility to pathogens. The probability of two plant hosts sharing a particular pest tends to decline with their phylogenetic distance (Gilbert et al., 2012), and it has been used to generate spatially explicit risk maps for host–pest associations (Robles‐Fernández & Lira‐Noriega, 2017). However, reversing the logic, if more closely related pathogens share traits to overcome host defences or have similar nutritional requirements, then they may be more likely to attack the same plant families. Explicitly incorporating trait information may improve these approaches further.

Species' traits are a proxy for growth, survival and reproductive performance (Laughlin & Messier, 2015) and underpin distribution, community structure, ecosystem function and evolutionary dynamics (McGill et al., 2006). Traits can also be important predictors of invasion (Moravcová et al., 2015) by influencing the outcome of biotic and abiotic interactions and species' relative fitness during transport, establishment and spread. Trait‐based invasion frameworks have been applied most often to plants, where growth rate, leaf morphology, plant size (Van Kleunen et al., 2010) and climatic tolerance (Gallagher et al., 2015) are often greater among invasive species compared to their non‐native, but non‐invasive, counterparts. Trait‐based analyses may have particular value in the context of horizon‐scanning approaches to biosecurity, which aim to identify invasive species considered medium or high priority threats in the near future. For example, the European Food Standards Agency use life‐history traits in preliminary screening to prioritise species for full pest risk assessments (PRA; EFSA PLH Panel (EFSA Panel on Plant Health), et al., 2018).

For plant pathogens, there is a scarcity of trait data and databases (Aguilar‐Trigueros et al., 2014), and many species are unknown to science at the point of emergence due to the vast under‐description of microbial distributions and diversity worldwide (Roy et al., 2017). For microbial taxa, the first conceptual trait‐based frameworks for understanding how distributions are filtered along abiotic gradients were developed only recently (Aguilar‐Trigueros et al., 2015; Crowther et al., 2014). The functional value of measured traits is not well understood, although empirical trait‐based studies of invasive pathogens are now beginning to accumulate. Spore morphology, optimum temperature for growth, the ability to disperse long distances, reproducing both sexually and asexually (McDonald & Linde, 2002; Philibert et al., 2011), attacking both forest and ornamental hosts (Santini et al., 2013) and cold tolerance (Redondo et al., 2018b) have been identified as potentially important predictors of invasiveness (McDonald & Linde, 2002).

We explore the relationship between traits and impact in the genus *Phytophthora* (‘plant‐destroyer’). *Phytophthora* species are oomycete plant pathogens with very severe economic impacts in the agricultural, horticultural and commercial forestry sectors, as well as major ecological impacts in the wider environment (Erwin & Ribeiro, 1996). Examples of recently invasive species include *Phytophthora ramorum*, the causal agent of Sudden Oak Death in the United States (Grünwald et al., 2008), ramorum blight in Europe (Werres & De Merlier, 2003) and sudden larch death in the UK (Webber et al., 2010). *Phytophthora cinnamomi* is the causal agent of large‐scale dieback in multiple woody hosts and in many regions, such as protected Kwongan vegetation in Southwest Australia (Burgess et al., 2017), and *Phytophthora* *x* *alni* has driven alder decline across Europe (Aguayo et al., 2014). The global diversity of *Phytophthora* is still unknown and may be two‐ to three‐fold higher than the nearly 200 known species (Scott et al., 2019). Among these undescribed *Phytophthora* species, future risks to plant health are unknown and potentially high. For example, *P. ramorum* is a notifiable pest in the EU, yet only appeared on quarantine pest lists following, rather than prior to emergence.

The UK Plant Health Risk Register identifies pest and pathogen attributes by which their risks should be ranked, including the number of hosts attacked, whether there are major impacts in the known range, host vulnerability and host mortality (Baker et al., 2014). Each of these attributes require existing knowledge about the behaviour, distribution and spread of the pest, which is unavailable at the point of emergence. By focussing on readily quantifiable biological traits available at the point of description (or even earlier) and phylogenetic relatedness to known high impact species, it may be possible to identify future potential threats much earlier in the invasion process.

We evaluate whether the traits of *Phytophthora* species could provide an early‐warning system for predicting whether newly described pathogen species are a future biosecurity threat. We aim to identify traits for future measurement that can support horizon‐scanning approaches (e.g. prioritising species for inclusion on national risk registers) and to highlight knowledge gaps and research priorities to improve trait‐based approaches.

## 2 MATERIALS AND METHODS

We developed a trait database of the morphological and physiological traits commonly used in taxonomic descriptions of *Phytophthora* (A. Perez‐Sierra & T. Burgess, unpubl. data) to quantify the role of traits, phylogeny and accumulation of knowledge (years since described) in driving interspecific variation in global impacts of *Phytophthora* species. All analyses were performed in R version 3.3.3 (R Core Team, 2017).

### 2.1 Global impact metrics

The number of countries reporting each species was compiled from publicly available databases (see Appendix S1) as a metric of the extent to which *Phytophthora* species have been introduced to new regions through anthropogenic pathways. Most inter‐country spread is via human‐mediated transport, so the number of countries reached should reflect species‐level differences in entrainment and successful transport within these pathways. Country‐level reports are a useful proxy for new *Phytophthora* detections because most plant health surveillance is reported at the level of National Plant Protection Organisation. We attempted to estimate global spatial extent as an impact metric, but spatially referenced records in our global *Phytophthora* database, as for many pathogen taxa, were too sparse to obtain a finer‐scale metric of within‐country spread. There was a significant positive correlation between country‐level occurrence and occupancy at 100 km cell resolution (*r = *0.67, *p* < 0.001), but country‐level reporting appears to be much more comprehensive across *Phytophthora* species. Even coarse resolution occupancy data (100 km) was limited to 95 species whereas country‐level data were available for 156 *Phytophthora* species.

We also extracted the maximum known latitude of *Phytophthora* species globally, as an absolute value. Where geo‐referenced data were available within a country, the absolute value of the highest latitude record was used. Following the approach used by Chaloner et al. (2020), where only country‐level data were available for a species, the centroid of the country with the highest absolute latitude was used as a proxy for the latitudinal limits of the species distribution. This metric was intended to reflect the distribution *Phytophthora* species in different climatic regions and interspecific variability in spread to higher latitudes. Latitudinal limits may also be less influenced by recording bias than number of countries, as recording intensity is probably greater towards latitudinal range edges (e.g. in northern temperate regions).

Host range was intended to capture the taxonomic breadth of *Phytophthora* species impacts on plants. As not all plant hosts are reported at species level, host range was measured as the number of plant families known to be infected by each *Phytophthora* species. Host associations for each *Phytophthora* species were collated from the Fungus‐Host Database of the U.S. National Fungus Collections (Farr & Rossman, 2018).

### 2.2 Trait data

We collated trait data for 179 *Phytophthora* species, of which 166 are formally described and 13 are provisionally named. Values for eight primary ecological trait values were extracted from species descriptions in the literature. The selected traits are hypothesised to have reproductive, survival, growth and/or dispersal functions. For example, oospores are dormant sexual propagules that persist in soil, asymptomatic plants and plant debris (reproduction, survival). Oospore wall index (the thickness of oospore walls relative to oospore volume) and minimum and optimum temperatures for growth are traits that influence tolerance to abiotic stressors, including temperature extremes and desiccation (survival). Asexual propagules called chlamydospores and hyphal swellings also function as alternative survival structures (survival, reproduction). Growth rate at optimum temperature measures vegetative growth in vitro (growth). Readily detached caducous sporangia become air‐borne and may germinate directly or differentiate to release further infective propagules termed zoospores, and proliferating sporangia produce successive rounds of propagules (dispersal). In Table 1, we describe these traits, their measurement and the hypothesised mechanisms by which they may confer greater success at one or more stages during the invasion process: arrival (via anthropogenic transport), establishment or spread.

**TABLE 1:** Eight primary ecological traits and two disease symptom traits of Phytophthora species with hypothesised mechanisms linking traits to higher global impacts (shaded grey: country‐level arrivals and host plant families affected)

Pathogen trait	Source	Format	Rationale	Countries reached (*introduction*)	Maximum latitude (*establishment*)	Host plant families (*impacts*)
Optimum (or minimum) temperatures for growth	Species descriptions	Numeric: °C	At higher latitudes, cold tolerance has been shown to facilitate pathogen establishment outside of nurseries in the wider environment (Redondo et al., 2018b). Cold stress is a limiting factor in the distribution of *P. cinnamomi* (Burgess et al., 2017) and latitude and mean temperature of the wettest quarter can predict regional variation in Australian *Phytophthora* diversity (Burgess et al., 2018)			
Oospores (resting sexual reproductive structures)	Species descriptions	Binary: 0 = no oospores produced, 1 = oospores produced	Sexual reproduction (oospore production) may confer greater potential for genetic diversity enabling host jumps and local adaptation in novel climates. Oospores can also persist in the absence of suitable environmental conditions and hosts facilitating survival Sterility (no oospores) could facilitate establishment in novel regions by enabling rapid clonal reproduction for well‐adapted strains			
Oospore wall index	Species descriptions	Numeric: ratio between the volume of the oospore wall and the volume of the entire oospore	Thicker walls relative to oospore volume should increase desiccation resistance and the environmental persistence of resting spores			
Growth rate at optimum temperature	Species descriptions	Numeric: mm/day	Fast‐growing species should have a competitive advantage, enhancing virulence and transmission rates (*establishment; dispersal*)			
Sporangial proliferation	Species descriptions	Binary: 0 = non‐proliferating; 1 = proliferating	Proliferating sporangia may enable more rapid production of zoospores (the infective propagules), which could facilitate establishment through dispersal			
Chlamydospores	Species descriptions	Binary: 0 = not observed; 1 = observed	Chlamydospores are thick‐walled, asexual resting structures and provide an alternative strategy to sexual reproduction			
Hyphal swellings	Species descriptions	Binary: 0 = not observed; 1 = observed	Hyphal swellings may be able to persist in plant tissue and offer an alternative resting structure			
Foliar disease symptoms	USDA National Fungus Collections	Binary: 0 = foliar disease not reported; 1 = foliar disease reported	Species that can sporulate in the foliar parts of plants may be able to disperse and spread rapidly by raining down on surrounding hosts and soil. Also linked to detectability			
Root disease symptoms	USDA National Fungus Collections	Binary: 0 = root disease not reported; 1 = root disease reported	Root‐infecting species are presumably soil‐borne and may be more easily transported globally with live plants			

In addition, we collated information on the ability to cause root and/or foliar disease for each *Phytophthora* species, based on disease notes in the U.S. National Fungus Collections (Farr & Rossman, 2018). These disease traits may capture variation in the detectability of different types of disease symptoms, but may also be ecologically important for invasion if they influence modes of dispersal (e.g. soil‐borne, air‐borne, water‐borne) and transmission among hosts (Table 1) or if species able to cause both root and foliar disease have more diverse mechanisms to overcome host defences.

### 2.3 Statistical analysis

We used phylogenetic generalised linear mixed models fitted in r package ‘brms’ (Bürkner, 2017; Appendix S2) to estimate the relationships between trait predictors and the two global impact metrics (the number of countries reached and host plant families impacted). Continuous traits were rescaled by dividing by two standard deviations (*SD*) of the mean value. This places binary and continuous predictors on approximately equal scales so parameter estimates are comparable in terms of relative effect sizes on the response (Gelman, 2008). We included time since description as a covariate to try to account for species‐level differences in recording effort and time to spread. We accounted for phylogenetic non‐independence among the species‐level observations, using a species‐specific random intercept with a mean of 0 and covariance defined by the phylogenetic relationships among *Phytophthora* species. We inferred species' phylogenetic relationships from an ITS6 phylogeny (T. Burgess, unpubl. data) including all 179 species in our trait database. A version of the analyses was also performed using a multi‐gene phylogeny (seven nuclear and four mitochondrial loci) for *Phytophthora* (Martin et al., 2014: TreeBASE S14595). This phylogeny includes fewer of the species in our trait database, but should provide more robust estimates of the phylogenetic relationships among these species (see Appendix S3). An observation‐level random intercept term was included to account for over‐dispersion in the counts of hosts and countries, relative to Poisson and binomial distributions, respectively (Harrison, 2014).

We fitted 2,560 candidate models comprising each possible subset of the ten trait predictors (2^10^ = 2,048), plus 512 models including an interaction term between foliar and root disease predictors (root disease × foliar disease) when both traits were present in the model to test whether species able to attack multiple plant parts reached more countries or had more hosts. Multi‐model comparison helps to reveal redundancy among correlated traits by indicating whether the effect of a trait on impact is robust to presence or absence of other predictors in the model, and was an attempt to understand the importance of predictors while avoiding the drawbacks of model averaging described by Cade (2015). Since our goal is to test how well the models can predict the number of countries reached, latitudinal limits and host families for newly described and emerging pathogens, we chose to rank these models based on out‐of‐sample predictive success using an information criterion derived from 10‐fold cross‐validation. The subset of top models for each impact metric was defined as all models within two information criterion (ΔIC) units of the best‐performing model. The direction, strength and significance of the trait effects were mapped for each model in the top subset to infer consistent and robust trait–impact relationships.

Within‐sample explanatory performance was quantified using the variance partitioning methods for generalised linear mixed effects models described in Nakagawa et al. (2017). We partition the variance explained for each global impact metric into marginal *R* ^2^ (the proportion of variance explained by the fixed effects of time since description and species' traits) and conditional *R* ^2^ (variance explained by fixed effects and phylogenetic relatedness). Phylogenetic signal in impact metrics is quantified as raw intra‐class correlation (ICC: the proportion of unexplained variance that is phylogenetically structured in the absence of fixed effects of traits and time since description) and adjusted intra‐class correlation (ICC_adj_: phylogenetically structured error after accounting for fixed effects). We report the lower and upper 95% credible intervals for all parameters, *R* ^2^ and ICC estimates.

### 2.4 Horizon scanning

We compared the observed and predicted values from the models for each species to identify species expected to have high impacts that have not yet occurred, based on their similar trait combinations and phylogenetic relatedness to well‐known high impact *Phytophthora* species, accounting for the level of accumulated knowledge (time since description). We ranked species based on the residuals from the best‐performing models for each impact metric to identify potential future biosecurity threats among recently described species (since 2010). Positive and negative residuals indicate species impacts are higher and lower, respectively, than the model predicts.

## 3 RESULTS

### 3.1 Impact metrics

Data on the number of countries reached, maximum latitude and number of host plant families were collated for 155, 144 and 167 *Phytophthora* species, respectively. The number of countries reporting each species ranged from 1 to 132 with a median of 3 countries per species. In all, 178 countries reported at least one *Phytophthora* species. Maximum absolute latitude ranged from 9.1° to 80.5° with a mean of 47.2°. The number of known host plant families ranged from 0 to 90 with a median of 2 per species.

### 3.2 Model performance

**FIGURE 1:**
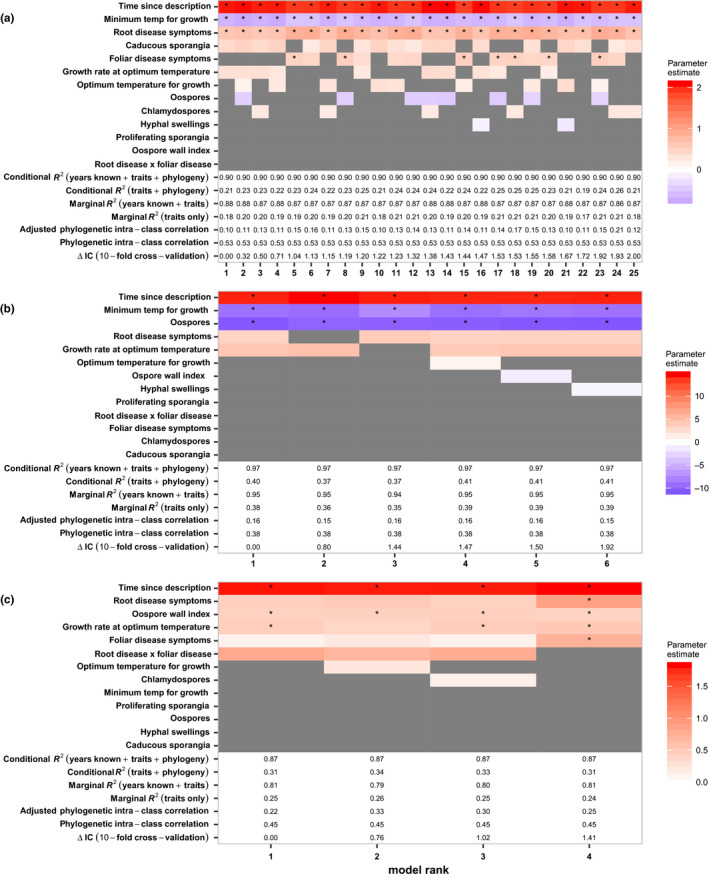
The best‐performing evolutionary trait‐based models of (a) number of countries reached (*n* = 117 *Phytophthora* species), (b) latitudinal limits (*n* = 123) and (c) number of host plant families attacked (*n* = 111). The best models were selected based on difference in information criterion, ΔIC, less than 2 from the highest‐ranked model. Significant effects (*) are defined as 95% credible intervals for a parameter estimate which do not overlap zero. Grey shading indicates trait predictors absent from the model. Red and blue colours indicate positive and negative parameter estimates, respectively, and depth of colour reflects relative effect size

In the highest‐ranked models, years since described, traits and phylogeny (Figure 1: conditional *R* ^2^) together explained 90% (LCI = 83, UCI = 94), 97% (72, 99) and 87% (79, 94) of interspecific variance in the number of countries (Figure 1a), maximum latitude (Figure 1b) and number of host families (Figure 1c) reported, respectively. Traits and phylogenetic relatedness (Figure 1: conditional $R_{\text{traits}+\text{phylogeny}}^{2}$) explained between 19% (8, 35) and 26% (12,4 5) of variance in the number of countries reached, between 37% (16, 62) and 41% (20, 65) of variance in maximum latitude and between 31% (14, 56) and 0.34% (18, 61) of variance in known host plant families. Traits alone (Figure 1: marginal $R_{\text{traits}}^{2}$) explained between 17% (6, 30)and 21% (11, 35) of variance in the number of countries reached, between 35% (14, 60) and 39% (18, 64) of variance in maximum latitude and between 24% (12, 38) and 26% (14, 39) of variance in number of host plant families. Phylogenetic intra‐class correlation (Figure 1) was strongest in the number of countries reached (53% [1, 87]) and slightly weaker in maximum latitude (38% [0, 50]) and known host families (45% [0, 89]). With the inclusion of species traits, phylogenetic signal (adjusted intra‐class correlation) decreased to between 10% (0, 57) and 24% (0, 74) in number of countries reached, to between 16% (0, 96) and 37% (2, 63) in maximum latitude and to between 22% (0, 78) and 33% (0, 83) in known host families.

### 3.3 Number of countries reached

Twenty‐five of the 2,560 trait‐based models of the number of countries reached by *Phytophthora* species were within 2 ΔIC units of the best model (Figure 1a). The odds of a country reporting a species increased 7.82 times (*β* = 2.06 [1.69, 2.43]) for every 69.75 years (2 *SD*s) since description. Minimum temperature for growth was present and significant in all 25 best‐ranked models. A 5.8℃ (2 *SD*s) increase in species minimum temperature for growth decreased the odds of a country reporting a species by a factor of 0.51 (*β* = −0.68 [−1.11, −0.25]. The ability to cause root disease was present and significant in all 25 best‐performing models of geographical extent and increased the odds of a country reporting a species by a factor of 1.87 (*β* = 0.62 [0.20, 1.06]). Foliar disease was present in 13 of the 25 best‐performing models, and was significant in seven of these models, increasing the odds of a country reporting a species by a factor of up to 1.70 (*β* = 0.53 [0.10, 0.96]). Caducous sporangia was present, but non‐significant, in 18 of the 25 best‐performing models and only when foliar disease was absent.

### 3.4 Maximum latitude

Six models of maximum latitude reached were within 2 ΔIC units of the best‐performing model (Figure 1b). The maximum latitude reported increased by 14.2° (9.08, 19.34) for every 69.75 years (2 *SD*s) since described. Minimum temperature for growth was present and significant in all 25 best‐ranked models. A 5.8℃ (2 *SD*s) increase in species minimum temperature for growth decreased maximum latitude reached by 9.0° (−13.73, −4.25). Oospore production was also present and significant in all six best‐performing models. Mean latitudinal limits were 10.7° (17.78, −3.6) lower for species producing oospores.

### 3.5 Host plant families

Four models of host range were within 2 ΔIC units of the best‐performing trait‐based model (Figure 1c). In the best‐performing model, the mean number of host families reported for a species increased 6.05 times (*β* = 1.80 [1.40, 2.19]) for every 69.75 years (2 *SD*s) since described. Root and foliar disease symptoms were both present in all four of the best‐performing models, but were non‐significant in three of these models. The ability to cause root disease and foliar disease increased the mean number of host plant families by 1.56 times (*β* = 0.45 [−0.27, 1.21]) and 1.13 times (*β* = 0.12 [−0.71, 1.02]), respectively. A positive, but non‐significant, interaction between root and foliar disease symptoms was apparent, but only when the main effects of each disease symptom were weaker (Figure 1c).

Oospore wall index and growth rate at optimum temperature were present in all four best‐performing models of number of known host plant families, and significant in 3 and 4 of those models, respectively. In the best‐performing model, the mean number of plant host families increased 1.56 times (*β* = 0.44 [0.06, 0.83]) for every 0.24 (2 *SD*) increase in oospore wall index and increased 1.60 times (*β* = 0.47 [0.01, 0.92]) with every 6.48 mm/day increase in growth rate.

### 3.6 Horizon scanning

**FIGURE 2:**
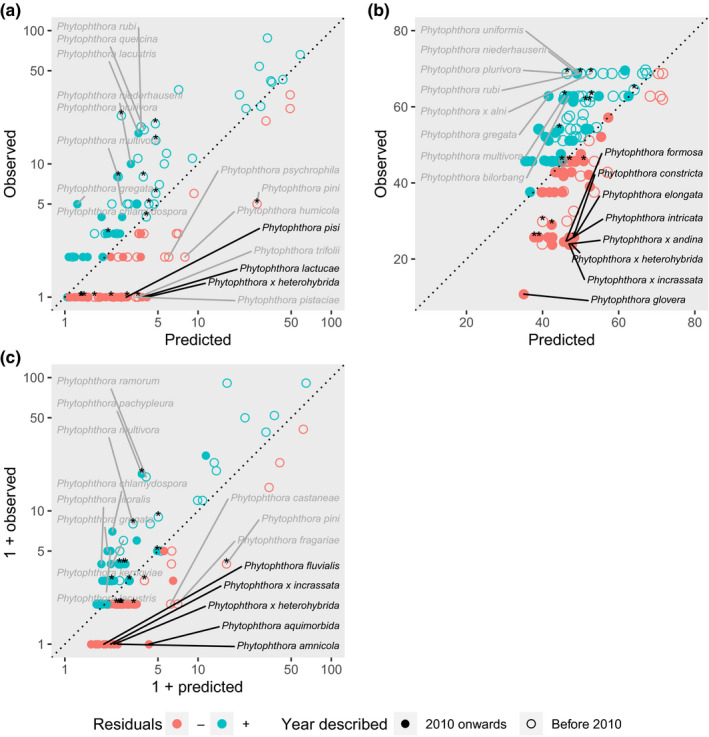
Horizon scanning for recently described *Phytophthora* species (filled circles text) with potential for future global impacts, using observed and predicted values for the best‐performing evolutionary trait‐based models of (a) number of countries reached, (b) latitudinal limits and (c) number of host plant families attacked. Labelled are the top eight *Phytophthora* species with observed impacts much lower (negative residuals; red circles) and higher (positive residuals; blue circles) than predicted by traits and phylogenetic position, adjusted for time since description. Recently described species are in bold. Asterisks denote recently resolved species, historically part of a species complex

Based on the best‐performing evolutionary trait‐based models, there are recently emerging *Phytophthora* species (described after 2010) reported in fewer countries (Figure 2a: *P. x heterohybrida*, *P. lactucae and P. pisi*), with lower observed latitudinal limits (Figure 2b: *Phytophthora glovera*, *P. x heterohybrida*, *P. x incrassata*, *P. constricta*, *P. elongata*, *P. x andina. P. flexuosa* and *P. intricata*) or with fewer known host families (Figure 2c: *P. aquimorbida*, *P. amnicola*, *P. x heterohybrida*, *P. x incrassata* and *P. fluvialis*) than would be predicted, given their phylogenetic and trait similarity to well‐known high impact species (for ranked lists, see Appendix S4). There were also species which have been known to science for longer among those with apparently under‐observed global impacts (e.g. *P. pini*, *P. fragariae* and *P. humicola*).

## 4 DISCUSSION

Our analyses of *Phytophthora* impacts demonstrate a framework using readily obtainable trait and phylogenetic data to produce ranked lists of future threats to plant health from emerging pathogens. We identify *Phytophthora* species closely related to or with similar traits to already high impact species, highlight potentially informative traits, and discuss knowledge gaps and research priorities to refine evolutionary trait‐based tools to support horizon‐scanning activities.

The primary methodology in horizon‐scanning approaches has been expert elicitation (Roy et al., 2015), but with limited information available on the behaviour, distribution and spread of recently described species, it can be difficult to resolve the relative threats posed by emerging pathogens. Integrating evolutionary trait‐based approaches into the consultation process has the potential to support the short listing of priority species and to generate more quantitative outputs from horizon‐scanning exercises.

### 4.1 Informative pathogen traits for horizon scanning

We compared the consistency of trait and phylogenetic effects on pathogen impacts between models using a single‐gene (ITS) and a multi‐gene phylogeny (Appendix S3). We only discuss results using the single‐gene phylogeny, as the importance of traits in analyses with a multi‐gene phylogeny was qualitatively the same, although the significance of primary ecological traits was less consistent, possibly due to reduced sample size and breadth of trait values in the 48 species in the multi‐gene phylogeny (Figure S2).

Disease symptoms were strong predictors of all three global impact metrics. Pathogens causing root disease symptoms may be more likely to be soil‐borne, potentially facilitating their global transport with ornamental plants (Migliorini et al., 2015). Foliar infection may facilitate rapid local spread by allowing propagules to become air‐borne or carried by rain splash. The rapid nationwide dissemination of a clone of *P. infestans* distributed on tomato plants via a major plant trader provides a good example of such spread (Fry et al., 2013) However, sporulation on leaves is also likely to increase detection and removal of diseased plants in transit. Incorporating trade flows into trait‐based early warning systems could help to clarify whether disease traits have a functional role in invasions. However, information about naturally occurring disease symptoms accumulates more slowly than data on primary ecological traits, limiting the use of such traits in early‐warning systems. Furthermore, disease symptoms may be conflated with pathogen detectability. Recent molecular surveys in water courses indicate *Phytophthora* species with a predominantly saprotrophic or opportunistic life history are more widespread than previously known (Hüberli et al., 2013). Their severe under‐reporting is presumably due to the rarity of disease symptoms.

Primary ecological traits predicting global impacts were minimum temperature for growth (number of countries reached, latitudinal limits), the absence of oospores (latitudinal limits), greater oospore wall indexes and faster growth rates (number of host families). Most *Phytophthora* growth assays start at refrigerator temperature (approx. 5°C), and focus on resolving optimal temperatures for growth. Despite the uncertainty associated with *Phytophthora* minimum temperature requirements, our results identified a signal of cold tolerance in predicting species' number of countries reached and latitudinal limits. Establishment of *Phytophthora* species outside of nurseries in Sweden was also positively associated with low temperature tolerance (Redondo et al., 2018b). Cooler, upland regions in Asia have been identified as part of the probable native range of a number of *Phytophthora* species that have invaded northern temperate forests (Brasier et al., 2010; Cleary et al., 2016; Jung et al., 2020). Our results highlight that more reliable measurements of minimum temperatures for growth should be a priority when characterising the thermal requirements of novel species but may also reflect that recording intensity tends be greater among temperate hosts and countries, compared to in tropical regions (Scott et al., 2019). Understanding the importance of this trait will depend on collaborative progress towards more comprehensive distribution databases that can be used to test whether trait‐mediated filtering of species establishment is consistent across climatic regions.

The absence of oospores was associated with species reaching greater latitudinal limits. Many clade six species, thought to be native to waterways at higher latitudes, share this trait, indicating oospore absence may be a good predictor of native, rather than invaded range and an alternative strategy to cold‐tolerance. The result is also consistent with water‐borne *Phytophthora* species being less sensitive to climatic gradients in temperature and precipitation, than species with soil‐borne life histories, due to greater buffering from extreme temperatures and desiccation for water‐borne propagules (Redondo et al., 2018a).

Greater oospore wall indexes were associated with broader host ranges and may confer greater desiccation resistance and long‐term survival of dormant propagules (Jung et al., 2013). Among fungal parasites, there is often a positive relationship between environmental persistence and virulence (Rafaluk‐Mohr, 2019). Remaining viable for longer in soils and asymptomatic hosts (Crone et al., 2013) should increase both encounter rates and successful transmission between novel hosts, both of which are necessary for host jumps. Faster growth rates were also associated with more host plant families and can confer a competitive advantage when colonising hosts (Alizon et al., 2009). Faster growth rates could also facilitate rapid adaptation and transmission to novel hosts through faster generation times (De Fine Licht, 2018).

Conditioning traits and time since description on phylogenetic position explained additional variance in global impact metrics, suggesting that using traits and phylogeny, jointly, may improve horizon scanning for future threats, because trait differences can lead to very different outcomes for closely related *Phytophthora* species. For example, *P. ramorum* and three lineages of *P. lateralis* (both clade 8c) may have co‐evolved in the same geographical origin, one with a broad host range (*P. ramorum*) and aerial spread, the other (*P. lateralis*) with narrow host range and predominantly root infecting (Vettraino et al., 2017). This suggests closely related species are capable of evolving very different hosts, traits and strategies. Even within species, lineages can evolve different traits to complete their life cycles (Brasier et al., 2012), indicating intraspecific trait and phylogenetic information could add further value to these approaches.

After adjusting for traits and years since described, residual phylogenetic intra‐class correlation in global impact metrics was relatively weak. Closely related species originate in the same geographical region and, therefore face the same geographical, biotic and environmental barriers, and have similar access to human transport pathways that may result in similar outcomes in invasion patterns. However, phylogenetic signal in range size may be weaker when some species have yet to fill their potential range (Dyer et al., 2016) or are under‐observed in their invaded range. The extent of this global disequilibrium and/or spatial recording bias in *Phytophthora* species distributions means the role of phylogenetic position may be underestimated in our models and could be a better predictor of future, rather than current, range size (see Appendix S3). Substantially reduced phylogenetic signal when including years since described and traits could also indicate taxonomic biases in *Phytophthora* recording and/or phylogenetically conserved traits. The latter is unlikely as both cold tolerance and oospore wall index are among the least phylogenetically conserved traits, but appear frequently in best‐performing models.

### 4.2 Refining evolutionary trait‐based approaches

Our models identify and rank recently described *Phytophthora* species with observed distributions (countries and latitudes reached) and host ranges substantially lower than predicted by their traits, phylogeny and time since description (Figure 2). These species may be those with the greatest potential for future global impacts. However, taxonomic biases in recording may also explain why some species are under‐ or over‐observed relative to model predictions. For example, *P. elongata* was previously part of the *P. citricola* species complex and will be under‐reported prior to the novel molecular tools that enabled this complex to be resolved. Over‐observed species include examples where isolates in some large culture collections have been reassigned to new taxa using molecular methods (*P. plurivora* and *P. multivora*; Burgess et al., 2009) and species that have, historically, been reported as a single species because of difficulties distinguishing hybrids (*P. x alni*). Modelling these species as complexes could improve model fit, but would discard valuable information about trait differences among poorly resolved species.

The eight species with global impacts much higher than predicted also include those previously targeted for increased surveillance effort. *P. rubi*, *P. ramorum*, *P. x alni* and *P. quercina* have all appeared on the European and Mediterranean Plant Protection Organisation (EPPO) alert list and may be disproportionately well‐reported compared to other species. *P. cinnamomi* is also probably one of the most well‐studied and highly surveyed *Phytophthora* species globally.

Addressing these taxonomic and spatial recording biases to provide more robust impact metrics will require greater knowledge of the native ranges of *Phytophthora* species to capture the full extent of *Phytophthora* distributions and host associations and the extent of niche overlap between native and invaded ranges. This will enable trait‐based approaches for pathogens to be incorporated into more spatially explicit frameworks for invasion risk assessment (already available for data‐rich taxa) including niche and spread models (Chapman et al., 2019; Engler et al., 2012). Novel meta‐barcoding methods and closer monitoring of invasions and finer‐grain distribution data, coupled with statistical methods to account for biases in recording effort could help to overcome differences in national spending on plant health surveillance (Scott et al., 2019). These data deficits highlight the need for collaborative centralised databases that compile data not only from different countries but also across the ornamental, agricultural and forestry sectors, alongside the wider environment and interceptions at ports‐of‐entry to get the most accurate picture of the behaviour of pathogens in terms of arrival, spread and host range. Such databases could also incorporate the potential ecological and economic impacts of plant pathogens into trait‐based early‐warning systems by promoting the sharing of data on pathogenicity tests (Wan & Liew, 2020) and the economic importance of individual hosts in different sectors (e.g. Dehnen‐Schmutz et al., 2007).

Predictions from evolutionary trait‐based models could be improved by broadening the traits measured for pathogens. Traits related to moisture requirements are not typically reported for *Phytophthora* species and could be especially relevant for predicting invasiveness (Crowther et al., 2014). Most growth assays focus solely on the growth rate of mycelium, but quantitative measures of sporangial, oospore and chlamydospore production at different temperatures could be a proxy for propagule pressure and the potential for spread in different climatic regions. While relatively few *Phytophthora* genomes have been sequenced, a number of genomic traits may also underpin evolutionary potential. Genome size and the number of families of particular classes of plant cell wall degrading enzymes (Kubicek et al., 2014) and pathogen effectors (Raffaele et al., 2010) have been implicated in pathogenicity of fungal and oomycete pathogens.

Linking traits to function is essential for the success of trait‐based predictions (Laughlin & Messier, 2015), but identifying trade‐offs and co‐selection among pathogen traits could be more informative about adaptive value than considering traits independently. A classic example in plants is the trade‐off between competitive ability and colonisation ability among plant species (Díaz et al., 2016). In the context of pathogen invasions, complimentary trait combinations could have synergistic effects if adaptive at different stages of invasion: transport, arrival, establishment, spread, impact and biosecurity interventions (Blackburn et al., 2011, 2014). For example, cold tolerance was an important predictor of number of countries reached and latitudinal limits, but not of host families. This could indicate that range expansion at higher latitudes is not sufficient to enable jumps to new host families and that additional mechanisms are needed to become both a widespread and host‐generalist pathogen. For example, *P. lateralis* is rather widespread but is still considered host‐specific (Hansen, 2015).

## 5 CONCLUSIONS

Our analyses evaluate an evolutionary trait‐based framework in which *Phytophthora* traits and phylogenetic relatedness explain up to 26%, 41% and 34% of variance in countries reached, latitudinal limits and plant host families attacked, respectively. Such trait‐based approaches could be used to support horizon‐scanning approaches for plant health biosecurity (Kamoun et al., 2015; Roy et al., 2017). We identify some recently described *Phytophthora* species with similar trait values and/or phylogenetic proximity to pathogens that have already been introduced to many countries (*P. x heterohybrida*, *P. lactucae and P. pisi*), have higher latitudinal limits (*Phytophthora glovera*, *P. x heterohybrida*, *P. x incrassata*, *P. constricta*, *P. elongata*, *P. x andina. P. flexuosa* and *P. intricata*) or have attacked hosts in many plant families (*P. aquimorbida*, *P. amnicola*, *P. x heterohybrida*, *P. x incrassata and P. fluvialis*. Priority traits to measure for newly arising species may be thermal tolerance, oospore wall index and growth rate at optimum temperature. However, our analyses also highlight significant knowledge gaps limiting the success of trait‐based approaches to horizon scanning: which pathogen traits have functional value in invasions; the extent of intraspecific variability in trait values; whether and how resource‐allocation trade‐offs and other evolutionary processes influence invasion success, and the poorly documented native and non‐native distributions of pathogens. This highlights the potential value of international and cross‐sectoral collaborations to provide centralised databases on pathogen distributions, environmental drivers, traits, phylogeny and invasion histories to support horizon scanning within plant health.

## AUTHORS' CONTRIBUTIONS

B.V.P. and L.J.B. conceived the study; L.J.B. compiled distribution and host data and wrote the manuscript with substantial contributions from all authors; T.I.B. provided the ITS phylogeny and A.P.‐S., A.H., B.H., P.S., N.W. and T.I.B. collated the trait data; P.S., D.E.L.C., T.I.B., G.H., B.H., A.P.‐S. and S.G. provided pathology and evolutionary expertise to inform the trait‐based hypotheses and assist with interpretation of results. All authors contributed critically to the drafts and gave final approval for publication.

## Supporting information

Supplementary Material

## Data Availability

Data available via the Zenodo open‐access repository https://doi.org/10.5281/zenodo.4081474 (Barwell et al., 2020).

## References

[jpe13820-bib-0001] Aguayo, J. , Elegbede, F. , Husson, C. , Saintonge, F. X. , & Marcais, B. (2014). Modeling climate impact on an emerging disease, the Phytophthora alni‐induced alder decline. Global Change Biology, 20(10), 3209–3221. 10.1111/gcb.12601 24729529

[jpe13820-bib-0002] Aguilar‐Trigueros, C. A. , Hempel, S. , Powell, J. R. , Anderson, I. C. , Antonovics, J. , Bergmann, J. , Cavagnaro, T. R. , Chen, B. , Hart, M. M. , Klironomos, J. , Petermann, J. S. , Verbruggen, E. , Veresoglou, S. D. , & Rillig, M. C. (2015). Branching out: Towards a trait‐based understanding of fungal ecology. Fungal Biology Reviews, 29(1), 34–41. 10.1016/j.fbr.2015.03.001

[jpe13820-bib-0003] Aguilar‐Trigueros, C. A. , Powell, J. R. , Anderson, I. C. , Antonovics, J. , & Rillig, M. C. (2014). Ecological understanding of root‐infecting fungi using trait‐based approaches. Trends in Plant Science, 19(7), 432–438. 10.1016/j.tplants.2014.02.006 24613596

[jpe13820-bib-0004] Alizon, S. , Hurford, A. , Mideo, N. , & Van Baalen, M. (2009). Virulence evolution and the trade‐off hypothesis: History, current state of affairs and the future. Journal of Evolutionary Biology, 22(2), 245–259. 10.1111/j.1420-9101.2008.01658.x 19196383

[jpe13820-bib-0005] Baker, R. H. A. , Anderson, H. , Bishop, S. , Macleod, A. , Parkinson, N. , & Tuffen, M. G. (2014). The UK Plant Health Risk Register: A tool for prioritizing actions. EPPO Bulletin, 44(2), 187–194. 10.1111/epp.12130

[jpe13820-bib-0006] Barwell, L. J. , Perez‐Sierra, A. , Henricot, B. , Harris, A. , Burgess, T. , Hardy, G. , Scott, P. , Williams, N. , Cooke, D. E. L. , Green, S. , Chapman, D. S. , & Purse, B. V. (2020). Phytophthora global impacts (Version v1.0.0). Zenodo, 10.5281/zenodo.4081474 PMC8048555 33883780

[jpe13820-bib-0007] Bebber, D. P. , Holmes, T. , & Gurr, S. J. (2014). The global spread of crop pests and pathogens. Global Ecology and Biogeography, 23(12), 1398–1407. 10.1111/geb.12214

[jpe13820-bib-0008] Blackburn, T. M. , Essl, F. , Evans, T. , Hulme, P. E. , Jeschke, J. M. , Kühn, I. , Kumschick, S. , Marková, Z. , Mrugała, A. , Nentwig, W. , Pergl, J. , Pyšek, P. , Rabitsch, W. , Ricciardi, A. , Richardson, D. M. , Sendek, A. , Vilà, M. , Wilson, J. R. U. , Winter, M. , … Bacher, S. (2014). A unified classification of alien species based on the magnitude of their environmental impacts. PLoS Biology, 12(5), e1001850. 10.1371/journal.pbio.1001850 24802715 PMC4011680

[jpe13820-bib-0009] Blackburn, T. M. , Pyšek, P. , Bacher, S. , Carlton, J. T. , Duncan, R. P. , Jarošík, V. , Wilson, J. R. U. , & Richardson, D. M. (2011). A proposed unified framework for biological invasions. Trends in Ecology & Evolution, 26(7), 333–339. 10.1016/J.TREE.2011.03.023 21601306

[jpe13820-bib-0010] Brasier, C. M. , Franceschini, S. , Vettraino, A. M. , Hansen, E. M. , Green, S. , Robin, C. , Webber, J. F. , & Vannini, A. (2012). Four phenotypically and phylogenetically distinct lineages in Phytophthora lateralis. Fungal Biology, 116(12), 1232–1249. 10.1016/j.funbio.2012.10.002 23245617

[jpe13820-bib-0011] Brasier, C. M. , Vettraino, A. M. , Chang, T. T. , & Vannini, A. (2010). *Phytophthora lateralis* discovered in an old growth *Chamaecyparis* forest in Taiwan. Plant Pathology, 59(4), 595–603. 10.1111/j.1365-3059.2010.02278.x

[jpe13820-bib-0012] Burgess, T. I. , McDougall, K. L. , Scott, P. M. , Hardy, G. E. S. , & Garnas, J. (2018). Predictors of *Phytophthora* diversity and community composition in natural areas across diverse Australian ecoregions. Ecography, 42, 1–14. 10.1111/ecog.03904

[jpe13820-bib-0013] Burgess, T. I. , Scott, J. K. , Mcdougall, K. L. , Stukely, M. J. C. , Crane, C. , Dunstan, W. A. , Brigg, F. , Andjic, V. , White, D. , Rudman, T. , Arentz, F. , Ota, N. , & Hardy, G. E. S. J. (2017). Current and projected global distribution of *Phytophthora cinnamomi*, one of the world's worst plant pathogens. Global Change Biology, 23(4), 1661–1674. 10.1111/gcb.13492 27596590

[jpe13820-bib-0014] Burgess, T. I. , Webster, J. L. , Ciampini, J. A. , White, D. , Hardy, G. E. S. , & Stukely, M. J. C. (2009). Re‐evaluation of phytophthora species isolated during 30 years of vegetation health surveys in Western Australia using molecular techniques. Plant Disease, 93(3), 215–223. 10.1094/Pdis-93-3-0215 30764178

[jpe13820-bib-0015] Bürkner, P.‐C. (2017). brms: An *R* package for Bayesian multilevel models using *Stan* . Journal of Statistical Software, 80(1), 1–28. 10.18637/jss.v080.i01

[jpe13820-bib-0070] Cade, B. S. (2015). Model averaging and muddled multimodel inferences. Ecology, 96, 2370–2382. 10.1890/14-1639.1 26594695

[jpe13820-bib-0016] Chaloner, T. M. , Gurr, S. J. , & Bebber, D. P. (2020). Geometry and evolution of the ecological niche in plant‐associated microbes. Nature Communications, 11(1), 1–9. 10.1038/s41467-020-16778-5 PMC7289842 32528123

[jpe13820-bib-0017] Chapman, D. , Pescott, O. L. , Roy, H. E. , & Tanner, R. (2019). Improving species distribution models for invasive non‐native species with biologically informed pseudo‐absence selection. Journal of Biogeography, 46(5), 1029–1040. 10.1111/jbi.13555

[jpe13820-bib-0018] Cleary, M. , Ghasemkhani, M. , Blomquist, M. , & Witzell, J. (2016). First Report of Phytophthora gonapodyides Causing Stem Canker on European Beech (*Fagus sylvatica*) in Southern Sweden. Plant Disease, 100(10), 2174. 10.1094/pdis-04-16-0468-pdn

[jpe13820-bib-0019] Crone, M. , McComb, J. A. , O'Brien, P. A. , & Hardy, G. E. S. J. (2013). Survival of *Phytophthora cinnamomi* as oospores, stromata, and thick‐walled chlamydospores in roots of symptomatic and asymptomatic annual and herbaceous perennial plant species. Fungal Biology, 117(2), 112–123. 10.1016/j.funbio.2012.12.004 23452949

[jpe13820-bib-0020] Crowther, T. W. , Maynard, D. S. , Crowther, T. R. , Peccia, J. , Smith, J. R. , & Bradford, M. A. (2014). Untangling the fungal niche: The trait‐based approach. Frontiers in Microbiology, 5(October), 1–12. 10.3389/fmicb.2014.00579 25400630 PMC4215788

[jpe13820-bib-0021] De Fine Licht, H. H. (2018). Does pathogen plasticity facilitate host shifts? PLoS Path, 14(5), e1006961. 10.1371/journal.ppat.1006961 PMC5933697 29723278

[jpe13820-bib-0022] Dehnen‐Schmutz, K. , Touza, J. , Perrings, C. , & Williamson, M. (2007). A century of the ornamental plant trade and its impact on invasion success. Diversity and Distributions, 13(5), 527–534. 10.1111/j.1472-4642.2007.00359.x

[jpe13820-bib-0023] Díaz, S. , Kattge, J. , Cornelissen, J. H. C. , Wright, I. J. , Lavorel, S. , Dray, S. , Reu, B. , Kleyer, M. , Wirth, C. , Colin Prentice, I. , Garnier, E. , Bönisch, G. , Westoby, M. , Poorter, H. , Reich, P. B. , Moles, A. T. , Dickie, J. , Gillison, A. N. , Zanne, A. E. , … Gorné, L. D. (2016). The global spectrum of plant form and function. Nature, 529(7585), 167–171. 10.1038/nature16489 26700811

[jpe13820-bib-0024] Dyer, E. E. , Franks, V. , Cassey, P. , Collen, B. , Cope, R. C. , Jones, K. E. , Şekercioğlu, Ç. H. , & Blackburn, T. M. (2016). A global analysis of the determinants of alien geographical range size in birds. Global Ecology and Biogeography, 25(11), 1346–1355. 10.1111/geb.12496

[jpe13820-bib-0037] EFSA PLH Panel (EFSA Panel on Plant Health) , Jeger, M. , Bragard, C. , Caffier, D. , Candresse, T. , Chatzivassiliou, E. , Dehnen‐Schmutz, K. , Grégoire, J.‐C. , Jaques Miret, J. A. , MacLeod, A. , Navajas Navarro, M. , Niere, B. , Parnell, S. , Potting, R. , Rafoss, T. , Rossi, V. , Urek, G. , Van Bruggen, A. , Van Der Werf, W. , … Gilioli, G. (2018). Guidance on quantitative pest risk assessment. EFSA Journal, 16(8), 5350. 10.2903/j.efsa.2018.5350 PMC7009646 32626011

[jpe13820-bib-0025] Engler, R. , Hordijk, W. , & Guisan, A. (2012). The MIGCLIM R package – Seamless integration of dispersal constraints into projections of species distribution models. Ecography, 35(10), 872–878. 10.1111/j.1600-0587.2012.07608.x

[jpe13820-bib-0026] Erwin, D. C. , & Ribeiro, O. K. (1996). Phytophthora diseases worldwide. APS Press.

[jpe13820-bib-0027] Farr, D. F. , & Rossman, A. Y. (2018). Fungal databases. U.S. National Fungus Collections, ARS, USDA.

[jpe13820-bib-0028] Fry, W. E. , McGrath, M. T. , Seaman, A. , Zitter, T. A. , McLeod, A. , Danies, G. , Small, I. M. , Myers, K. , Everts, K. , Gevens, A. J. , Gugino, B. K. , Johnson, S. B. , Judelson, H. , Ristaino, J. , Roberts, P. , Secor, G. , Seebold, K. , Snover‐Clift, K. , Wyenandt, A. , … Smart, C. D. (2013). The 2009 late blight pandemic in the eastern United States – Causes and results. Plant Disease, 97(3), 296–306. 10.1094/PDIS-08-12-0791-FE 30722376

[jpe13820-bib-0029] Gallagher, R. V. , Randall, R. P. , & Leishman, M. R. (2015). Trait differences between naturalized and invasive plant species independent of residence time and phylogeny. Conservation Biology, 29(2), 360–369. 10.1111/cobi.12399 25369762 PMC4405095

[jpe13820-bib-0030] Gallardo, B. , Zieritz, A. , Adriaens, T. , Bellard, C. , Boets, P. , Britton, J. R. , Newman, J. R. , van Valkenburg, J. L. C. H. , & Aldridge, D. C. (2016). Trans‐national horizon scanning for invasive non‐native species: A case study in western Europe. Biological Invasions, 18(1), 17–30. 10.1007/s10530-015-0986-0

[jpe13820-bib-0031] Gelman, A. (2008). Scaling regression inputs by dividing by two standard deviations. Statistics in Medicine, 27, 2865–2873. 10.1002/sim 17960576

[jpe13820-bib-0032] Gilbert, G. S. , Magarey, R. , Suiter, K. , & Webb, C. O. (2012). Evolutionary tools for phytosanitary risk analysis: Phylogenetic signal as a predictor of host range of plant pests and pathogens. Evolutionary Applications, 5(8), 869–878. 10.1111/j.1752-4571.2012.00265.x 23346231 PMC3552404

[jpe13820-bib-0033] Grünwald, N. J. , Goss, E. M. , & Press, C. M. (2008). *Phytophthora ramorum*: A pathogen with a remarkably wide host range causing sudden oak death on oaks and ramorum blight on woody ornamentals. Molecular Plant Pathology, 9(6), 729–740. 10.1111/j.1364-3703.2008.00500.x 19019002 PMC6640315

[jpe13820-bib-0034] Hansen, E. M. (2015). *Phytophthora* species emerging as pathogens of forest trees. Current Forestry Reports, 1(1), 16–24. 10.1007/s40725-015-0007-7

[jpe13820-bib-0035] Harrison, X. A. (2014). Using observation‐level random effects to model overdispersion in count data in ecology and evolution. PeerJ, 2, e616. 10.7717/peerj.616 25320683 PMC4194460

[jpe13820-bib-0036] Hüberli, D. , Hardy, G. E. S. J. , White, D. , Williams, N. , & Burgess, T. I. (2013). Fishing for *Phytophthora* from Western Australia's waterways: A distribution and diversity survey. Australasian Plant Pathology, 42(3), 251–260. 10.1007/s13313-012-0195-6

[jpe13820-bib-0038] Jung, T. , Colquhoun, I. J. , & Hardy, G. E. S. J. (2013). New insights into the survival strategy of the invasive soilborne pathogen *Phytophthora cinnamomi* in different natural ecosystems in Western Australia. Forest Pathology, 43(4), 266–288. 10.1111/efp.12025

[jpe13820-bib-0039] Jung, T. , Scanu, B. , Brasier, C. , Webber, J. , Milenković, I. , Corcobado, T. , Tomšovský, M. , Pánek, M. , Bakonyi, J. , Maia, C. , Bačová, A. , Raco, M. , Rees, H. , Pérez‐Sierra, A. , & Horta Jung, M. (2020). A survey in natural forest ecosystems of Vietnam reveals high diversity of both new and described phytophthora taxa including *P. ramorum* . Forests, 11(1), 93. 10.3390/f11010093

[jpe13820-bib-0040] Kamoun, S. , Furzer, O. , Jones, J. D. G. , Judelson, H. S. , Ali, G. S. , Dalio, R. J. D. , Roy, S. G. , Schena, L. , Zambounis, A. , Panabières, F. , Cahill, D. , Ruocco, M. , Figueiredo, A. , Chen, X.‐R. , Hulvey, J. , Stam, R. , Lamour, K. , Gijzen, M. , Tyler, B. M. , … Govers, F. (2015). The Top 10 oomycete pathogens in molecular plant pathology. Molecular Plant Pathology, 16(4), 413–434. 10.1111/mpp.12190 25178392 PMC6638381

[jpe13820-bib-0041] Kubicek, C. P. , Starr, T. L. , & Glass, N. L. (2014). Plant cell wall‐degrading enzymes and their secretion in plant‐pathogenic fungi. Annual Review of Phytopathology, 52, 427–451. 10.1146/annurev-phyto-102313-045831 25001456

[jpe13820-bib-0042] Laughlin, D. C. , & Messier, J. (2015). Fitness of multidimensional phenotypes in dynamic adaptive landscapes. Trends in Ecology & Evolution, 30(8), 487–496. 10.1016/j.tree.2015.06.003 26122484

[jpe13820-bib-0043] Lowe, S. , Browne, M. , Boudjelas, S. , & De Poorter, M. (2000). 100 of the world's worst invasive alien species a selection from the global invasive species database. The Invasive Species Specialist Group (ISSG) a specialist group of the Species Survival Commission (SSC) of the World Conservation Union (IUCN).

[jpe13820-bib-0044] Martin, F. N. , Blair, J. E. , & Coffey, M. D. (2014). A combined mitochondrial and nuclear multilocus phylogeny of the genus *Phytophthora* . Fungal Genetics and Biology, 66, 19–32. 10.1016/j.fgb.2014.02.006 24603058

[jpe13820-bib-0045] McDonald, B. A. , & Linde, C. (2002). Pathogen population genetics, evolutionary potential, and durable resistance. Annual Review of Phytopathology, 40(1), 349–379. 10.1146/annurev.phyto.40.120501.101443 12147764

[jpe13820-bib-0046] McGill, B. J. , Enquist, B. J. , Weiher, E. , & Westoby, M. (2006). Rebuilding community ecology from functional traits. Trends in Ecology & Evolution, 21(4), 178–185. 10.1016/j.tree.2006.02.002 16701083

[jpe13820-bib-0047] Migliorini, D. , Ghelardini, L. , Tondini, E. , Luchi, N. , & Santini, A. (2015). The potential of symptomless potted plants for carrying invasive soilborne plant pathogens. Diversity and Distributions, 21(10), 1218–1229. 10.1111/ddi.12347

[jpe13820-bib-0048] Moravcová, L. , Pyšek, P. , Jarošík, V. , & Pergl, J. (2015). Getting the right traits: Reproductive and dispersal characteristics predict the invasiveness of herbaceous plant species. PLoS ONE, 10(4), 1–16. 10.1371/journal.pone.0123634 PMC4407890 25906399

[jpe13820-bib-0049] Nakagawa, S. , Johnson, P. , & Schielzeth, H. (2017). The coefficient of determination *R* ^2^ and intra‐class correlation coefficient from generalized linear mixed‐effects models revisited and expanded. Journal of the Royal Society Interface, 14, 20170213. 10.1098/rsif.2017.0213 28904005 PMC5636267

[jpe13820-bib-0050] Philibert, A. , Desprez‐Loustau, M.‐L. , Fabre, B. , Frey, P. , Halkett, F. , Husson, C. , Lung‐Escarmant, B. , Marçais, B. , Robin, C. , Vacher, C. , & Makowski, D. (2011). Predicting invasion success of forest pathogenic fungi from species traits. Journal of Applied Ecology, 48(6), 1381–1390. 10.1111/j.1365-2664.2011.02039.x

[jpe13820-bib-0051] Pimentel, D. , Zuniga, R. , & Morrison, D. (2005). Update on the environmental and economic costs associated with alien‐invasive species in the United States. Ecological Economics, 52(3), 273–288. 10.1016/j.ecolecon.2004.10.002

[jpe13820-bib-0052] R Core Team . (2017). R: A language and environment for statistical computing. R Foundation for Statistical Computing.

[jpe13820-bib-0053] Rafaluk‐Mohr, C. (2019). The relationship between parasite virulence and environmental persistence: A meta‐analysis. Parasitology, 146(7), 897–902. 10.1017/S0031182019000015 30777585

[jpe13820-bib-0054] Raffaele, S. , Farrer, R. A. , Cano, L. M. , Studholme, D. J. , MacLean, D. , Thines, M. , Jiang, R. H. Y. , Zody, M. C. , Kunjeti, S. G. , Donofrio, N. M. , Meyers, B. C. , Nusbaum, C. , & Kamoun, S. (2010). Genome evolution following host jumps in the Irish potato famine pathogen lineage. Science, 330(6010), 1540–1543. 10.1126/science.1193070 21148391

[jpe13820-bib-0055] Redondo, M. A. , Boberg, J. , Stenlid, J. , & Oliva, J. (2018a). Contrasting distribution patterns between aquatic and terrestrial *Phytophthora* species along a climatic gradient are linked to functional traits. The ISME Journal, 12, 2967–2980. 10.1038/s41396-018-0229-3 30072746 PMC6246556

[jpe13820-bib-0056] Redondo, M. A. , Boberg, J. , Stenlid, J. , & Oliva, J. (2018b). Functional traits associated with the establishment of introduced *Phytophthora* spp. Swedish forests. Journal of Applied Ecology, 55(3), 1538–1552. 10.1111/1365-2664.13068

[jpe13820-bib-0057] Robles‐Fernández, Á. L. , & Lira‐Noriega, A. (2017). Combining phylogenetic and occurrence information for risk assessment of pest and pathogen interactions with host plants. Frontiers in Applied Mathematics and Statistics, 3, 17. 10.3389/fams.2017.00017

[jpe13820-bib-0058] Roy, H. E. , Adriaens, T. , Aldridge, D. C. , Bacher, S. , Bishop, J. D. D. , Blackburn, T. M. , Branquart, E. , Brodie, J. , Carboneras, C. , Cottier‐Cook, E. J. , Copp, G. H. , Dean, H. J. , Eilenberg, J. , Essl, F. , Gallardo, B. , García Criado, M. , García‐Berthou, E. , Genovesi, P. , Hulme, P. , … Zenetos, A. (2015). Invasive alien species – Prioritising prevention efforts through horizon scanning ENV.B.2/ETU/2014/0016. European Commission.

[jpe13820-bib-0059] Roy, H. E. , Bacher, S. , Essl, F. , Adriaens, T. , Aldridge, D. C. , Bishop, J. D. D. , Blackburn, T. M. , Branquart, E. , Brodie, J. , Carboneras, C. , Cottier‐Cook, E. J. , Copp, G. H. , Dean, H. J. , Eilenberg, J. , Gallardo, B. , Garcia, M. , García‐Berthou, E. , Genovesi, P. , Hulme, P. E. , … Rabitsch, W. (2019). Developing a list of invasive alien species likely to threaten biodiversity and ecosystems in the European Union. Global Change Biology, 25(3), 1032–1048. 10.1111/gcb.14527 30548757 PMC7380041

[jpe13820-bib-0060] Roy, H. E. , Hesketh, H. , Purse, B. V. , Eilenberg, J. , Santini, A. , Scalera, R. , Stentiford, G. D. , Adriaens, T. , Bacela‐Spychalska, K. , Bass, D. , Beckmann, K. M. , Bessell, P. , Bojko, J. , Booy, O. , Cardoso, A. C. , Essl, F. , Groom, Q. , Harrower, C. , Kleespies, R. , … Dunn, A. M. (2017). Alien pathogens on the horizon: Opportunities for predicting their threat to wildlife. Conservation Letters, 10(4), 476–483. 10.1111/conl.12297

[jpe13820-bib-0061] Roy, H. E. , Peyton, J. , Aldridge, D. C. , Bantock, T. , Blackburn, T. M. , Britton, R. , Clark, P. , Cook, E. , Dehnen‐Schmutz, K. , Dines, T. , Dobson, M. , Edwards, F. , Harrower, C. , Harvey, M. C. , Minchin, D. , Noble, D. G. , Parrott, D. , Pocock, M. J. O. , Preston, C. D. , … Walker, K. J. (2014). Horizon scanning for invasive alien species with the potential to threaten biodiversity in Great Britain. Global Change Biology, 20(12), 3859–3871. 10.1111/gcb.12603 24839235 PMC4283593

[jpe13820-bib-0062] Santini, A. , Ghelardini, L. , De Pace, C. , Desprez‐Loustau, M. L. , Capretti, P. , Chandelier, A. , Cech, T. , Chira, D. , Diamandis, S. , Gaitniekis, T. , Hantula, J. , Holdenrieder, O. , Jankovsky, L. , Jung, T. , Jurc, D. , Kirisits, T. , Kunca, A. , Lygis, V. , Malecka, M. , … Stenlid, J. (2013). Biogeographical patterns and determinants of invasion by forest pathogens in Europe. New Phytologist, 197(1), 238–250. 10.1111/j.1469-8137.2012.04364.x 23057437

[jpe13820-bib-0063] Scott, P. , Bader, M.‐K.‐F. , Burgess, T. , Hardy, G. , & Williams, N. (2019). Global biogeography and invasion risk of the plant pathogen genus Phytophthora. Environmental Science & Policy, 101, 175–182. 10.1016/J.ENVSCI.2019.08.020

[jpe13820-bib-0064] Shine, C. , Kettunen, M. , Genovesi, P. , Essl, F. , Gollasch, S. , Rabitsch, W. , Scalera, R. , Starfinger, U. , & ten Brink, P. (2010). Assessment to support continued development of the EU strategy to combat invasive alien species. Final Report for the European Commission.

[jpe13820-bib-0065] Van Kleunen, M. , Weber, E. , & Fischer, M. (2010). A meta‐analysis of trait differences between invasive and non‐invasive plant species. Ecology Letters, 13(2), 235–245. 10.1111/j.1461-0248.2009.01418.x 20002494

[jpe13820-bib-0066] Vettraino, A. M. , Brasier, C. M. , Webber, J. F. , Hansen, E. M. , Green, S. , Robin, C. , Tomassini, A. , Bruni, N. , & Vannini, A. (2017). Contrasting microsatellite diversity in the evolutionary lineages of *Phytophthora lateralis* . Fungal Biology, 121, 112–126. 10.1016/J.FUNBIO.2016.10.002 28089043

[jpe13820-bib-0067] Wan, J. S. H. , & Liew, E. C. Y. (2020). Genus‐level change in aggressiveness with continuous invasions: A phylogenetically‐informed Bayesian quantile regression. Biological Invasions, 22(6), 1931–1946. 10.1007/s10530-020-02229-1

[jpe13820-bib-0068] Webber, J. F. , Mullett, M. , & Brasier, C. M. (2010). Dieback and mortality of plantation Japanese larch (*Larix kaempferi*) associated with infection by *Phytophthora ramorum* . New Disease Reports, 22, 19. 10.1071/AP07088

[jpe13820-bib-0069] Werres, S. , & De Merlier, D. (2003). First detection of *Phytophthora ramorum* mating type A2 in Europe. Plant Disease, 87(10), 1266. 10.1094/PDIS.2003.87.10.1266C 30812744

