# Supplementary material for: Evolutionary trait‐based approaches for predicting future global impacts of plant pathogens in the genus Phytophthora
Source: J Appl Ecol. 2020 Dec 23;58(4):718–30. doi: 10.1111/1365-2664.13820 (PMC8048555; doi:10.1111/1365-2664.13820)
Supplement: Supplementary file 1 — Supplementary Material [file JPE-58-718-s001.docx]

**Supporting Information**

**Evolutionary trait-based approaches for predicting global impacts of plant pathogens in the genus *Phytophthora***

**Appendix S1** Global *Phytophthora* distribution data

We compiled distribution data comprising isolations of pathogens into culture as well as DNA-based identifications using techniques such as PCR and sequencing, quantitative real-time PCR and meta-barcoding. Distribution data for *Phytophthora* species (Fig. S1) were sourced from from CAB Abstracts (n = 16247, CABI, 2018a) using the search term "title:(Phytophthora)". The geographic location was extracted from the CAB Abstract full record and the focal *Phytophthora* species were identified by text mining the CAB Abstract titles for species names. Country-level *Phytophthora* reports were also extracted from CABI Invasive Species Compendium datasheets (n = 387, CABI, 2018b) using the search term “Phytophthora&types=7,17,19”. Distribution data from the EPPO Global Database and EPPO Reporting Service (n = 1129, EPPO, 2018) were extracted from online distribution tables via R. Data from the GBIF database (n = 418, GBIF occurrence download https://doi.org/10.15468/dl.dqsr1u) was queried using the R package ‘rgbif’ (Chamberlain & Boettiger, 2017). Country-level distribution tables for *Phytophthora* species were extracted from online distribution tables at the DAISIE European Invasive Alien Species Gateway (DAISIE, 2018) and from NCBI Biosample (https://www.ncbi.nlm.nih.gov/biosample) records using the R package ‘rvest’ (Wickham, 2016). Distribution data were also obtained from the Index Fungorum (www.indexfungorum.org) database API using the R package ‘taxize’ (Chamberlain & Szocs, 2013) and from the Fungal Collection of the CBS strain database (<http://www.westerdijkinstitute.nl/Collections>). A small number of plant health interceptions in the dataset were excluded, as our global spread metric was designed to capture impactful, and not transient or failed, invasions. The data were supplemented with our own geo-referenced database of *Phytophthora* records collated through an international network of 107 expert pathologists.

**
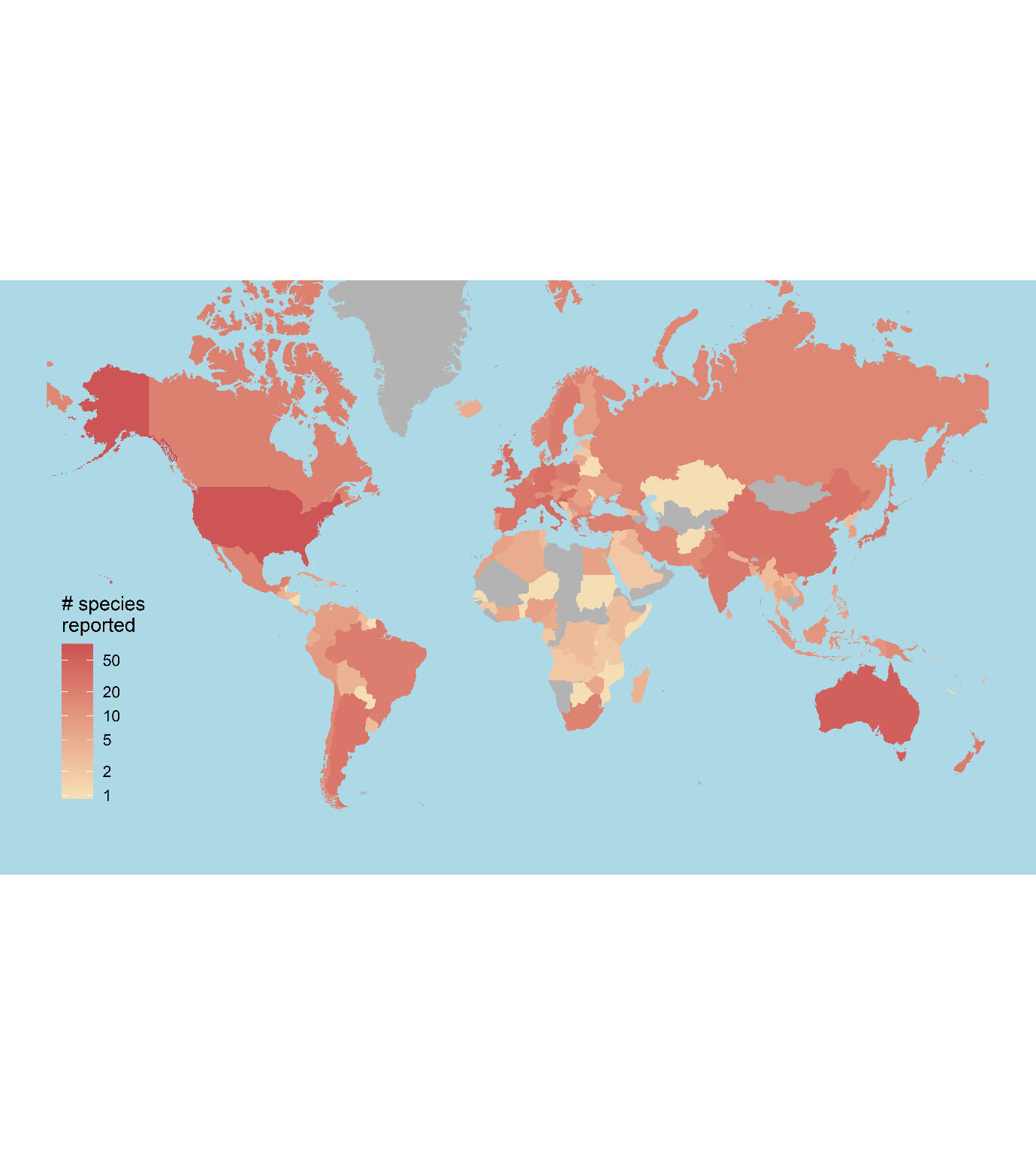
**

**Figure S1** Global reporting of *Phytophthora* species by country.

**Appendix S2**

Bayesian phylogenetic generalised linear mixed models of global impacts

Models of global impacts were specified as,

$g\left( y_{i} \right)= \beta_{0}+ \sum_{h=1}^{j} \beta_{h}x_{h_{i}}+\beta years known + \alpha_{i}+ e_{i},$(Eq. A1)

$\alpha_{i} \sim Gaussian(0, \sigma_{\alpha}^{2})$,

$e_{i} \sim Gaussian(0, \sigma_{e}^{2})$,

where g() is the appropriate link function relating the trait predictors to the impact metric on the linear predictor scale and $y_{i}$is the expected impact of species *i*. The number of known host families for the *Phytophthora* species was modelled as a Poisson response with log link *y*_i_ ~ *Poisson*(λ_i_) where $\ln\left( \lambda_{i} \right)$is the expected value on the log link scale. The number of countries reached was modelled as a binomial response with logit link, $y_{i} \sim binomial\left( n_{i}, p_{i} \right)$ where the number of trials was specified as the total number of countries reporting 1 or more *Phytophthora* species in our species-by-country occurrence database ($n_{i}=179$) and $\mathrm{logit} \left( p_{i} \right)$is the expected probability on the logit scale. $\beta_{0}$ is the overall intercept, $\beta_{h}$ is the estimated coefficient for trait *h* and $x_{h_{i}}$is the observed value of trait *h* for species *i*. Phylogenetic non-independence and over-dispersion of model residuals relative to model assumptions and can both lead to biased parameter estimates, associated standard errors, and the variance components attributable to main effects (Harrison, 2014). Therefore, we account for phylogenetic non-independence among the species-level observations using a unique species-level intercept, $\alpha_{i}$, with mean of zero and variance among species, $\sigma_{\alpha}^{2}$, derived from their phylogenetic position, which also allows us to partition the variance explained by traits and phylogenetic relationships.

We include an observation-level random effect, $e_{i}$, with mean of zero and variance,$\sigma_{e}^{2}$, to explicitly account for overdispersion when estimating the model parameters (Harrison, 2014).

We specified weak normal priors with a mean of zero and a standard deviation of 10 for $\beta_{h}$,and a mean of zero and a standard deviation of 50 for $\beta_{0}$. For the standard deviation of the phylogenetically structured random effects, $\sigma_{\alpha}^{2}$ , and the observation level random effect, $\sigma_{e}^{2}$, we used a weakly informative half student-t prior with 4 degrees of freedom and scale parameter of 1 to improve sampling time and to reflect that the standard deviation must be positive (Piironen & Vehtari, 2015).

We ranked the out-of-sample predictive performance of the 2560 models using an information criterion based on ten-fold cross-validation, which randomly partitions the data into ten subsets and then refits the model ten times, each time leaving out a different subset with which to compare the observed and predicted impacts. An information criterion for each model is derived from the summed expected log pointwise predictive density (ELPD) for the predicted data (Gelman, Hwang, & Vehtari, 2014) and the effective number of parameters in the model. The ELPD is multiplied by -2 to place it on the deviance scale, akin to other information criteria like AIC and DIC.

Leave-one-out cross validation would have been preferable but was not computationally feasible, as each of the 2560 models would have to be refitted between 48 and 123 times, depending on the number of species with available trait and impact data.

Sterile *Phytophthora* species do not produce oospores and have no values for the oospore wall index trait. These species were assigned the mean of oospore wall index across species to minimise their influence on model parameter estimates, while avoiding the exclusion of all sterile species from the analyses.

To explore multicollinearity between trait predictors, we calculated variance inflation factors for each trait using the function vif in R package ‘car’. A variance inflation factor of 1 indicates no multicollinearity, while values of greater than 5 are considered indicative of redundancy among predictors. For linear models including all trait predictors, generalised variance inflation factors were less than 4 for all trait predictors (Table S1). All traits were therefore retained in the full model.

Table S1 Variance inflation factors (VIF) for each of the trait predictors used to model global impact metrics.

| **Trait** | **Number of countries reached** | **Latitudinal limits** | **Number of known host families** |
| --- | --- | --- | --- |
| Foliar disease | 3.71 | 3.60 | 3.99 |
| Foliar disease x root disease | 3.16 | 3.04 | 3.45 |
| optimum temperature for growth | 2.68 | 2.63 | 2.68 |
| root disease | 2.54 | 2.42 | 2.63 |
| minimum temperature for growth | 1.93 | 1.83 | 1.94 |
| proliferating sporangia | 1.73 | 1.64 | 1.76 |
| Caducous sporangia | 1.61 | 1.60 | 1.62 |
| Growth rate at optimum temperature | 1.57 | 1.65 | 1.56 |
| Oospore presence | 1.24 | 1.24 | 1.25 |
| Years since described | 1.23 | 1.22 | 1.23 |
| Oospore wall index | 1.22 | 1.22 | 1.23 |
| Hyphal swellings | 1.20 | 1.18 | 1.20 |
| Chlamydospores | 1.07 | 1.07 | 1.08 |

**Appendix S3**

*Phytophthora* phylogenetic relationships

Phylogenetic non-independence in the residuals of fitted models can lead to under-estimates of confidence intervals for parameter estimates and a greater risk of type 1 errors in inference. To account for shared phylogenetic history when estimating the effects of traits on *Phytophthora* global impacts, we included a phylogenetically structured species-specific random intercept, constrained by the species-by-species phylogenetic covariance matrix.

Our ITS-based phylogeny (T. Burgess, unpublished data) includes all 179 species in the trait database. A single representative was included for each described and provisional species. Where available this is the sequence of the type isolate. If the type isolate was not available, sequence for several isolates was downloaded from Genbank and aligned and the consensus sequence extracted to obtain a representative sequence. ITS sequences were aligned in Geneious R9 and Bayesian analysis conducted using a General Time Reversible (GTR) substitution model and gamma rate variation. The Markov Chain Monte Carlo analysis of four chains started from random tree topology and lasted 1 million generations; burnin was set at 200,000. The resultant tree was saved in Newick format and exported for further analyses.

A single-gene phylogeny is typically less well-resolved, especially in the deeper nodes of the tree, and may have less statistical support than a phylogeny that considers phylogenetic relationships based on multiple loci (Gontcharov, Marin, & Melkonian, 2003). Multichotomies in the ITS phylogeny were resolved by transformation into a series of dichotomies each with one or more branches of length zero using the function *multi2di* in R package ape (Paradis, Claude, & Strimmer, 2004). The Martin *et al.*, (2014) phylogeny was rooted by removing one of the two outgroups. Both phylogenetic trees were converted from non-ultrametric to ultrametric, using a semi-parametric method based on penalized likelihood with a smoothing parameter of 1 (*chronopl* in R package ‘ape’: Paradis *et al.*, 2004).

Inverse covariance matrices were derived from both phylogenetic trees (*inverseA* in R package MCMCglmm: Hadfield, 2010) and then converted to covariance matrices for use in ‘brms’ models (Bürkner, 2017).

The single gene phylogeny includes all species in the trait database and enabled us to incorporate all species with complete trait and impacts data and into our analyses (n = 118 for geographic extent models; n = 113 for host range models). The analyses with the multi-gene phylogeny in Martin *et al.*, (2014) have substantially less power as the phylogeny includes only 48 species with complete trait data for modelling. However, the phylogenetic relationships among species should be better resolved. This subset of species are generally well-recorded with good species-level knowledge, whilst our complete dataset includes many recently described species with less well understood distributions and host ranges.

Consistent with models using the single gene phylogeny, root disease and/or foliar disease symtoms appeared frequently in the best-performing models of geographic extent, latitudinal limits and host range when using the multi-gene phylogeny (Fig. S1). The strong effect of minimum temperature for growth on number of countries and maximum latitudes reached was also consistent between models using single gene and multi-gene phylogenies. In models of host range, a signal of optimum temperature for growth was apparent, which was not detected in models using the single gene phylogeny. (Fig. S1c). This reversal of importance may be due this subset of species being those known to science for longer and with greater knowledge of their host associations. Many warm-adapted *Phytophthora* species known from the tropics are well-studied agricultural pests. They are typically heterothallic (capable of outcrossing) and both mating types are usually present, suggesting they may maintain high levels of genetic diversity in response to the high local host diversity in the tropics (Jung et al., 2011), perhaps enabling these species to exploit a greater range of hosts (Wang, Coffey, De Maio, & Goss, 2020). The signal of warm-adapted species having broader host ranges may be diluted among the much larger sample of species included the single gene analysis, where the host ranges of more recently described *Phytophthora* species found in surveys of natural environments in the tropics are typically poorly known (e.g. Jung et al., 2020). Alternatively the inconsistent effect of optimum temperature for growth between the single- and multi-gene analyses may reflect that optimum temperature for growth has the greatest VIF after disease symptom traits, indicating some moderate multicollinearity with other traits (Table S1). Oospore wall index was absent from best-performing models of host range using the multi-gene phylogeny possibly due the restricted sample size when using the multigene phylogeny. By contrast, oospore wall index was present and significant in all but two of the best-performing models of host range using the single gene phylogeny.

Our analysis with the multi-gene phylogeny (Fig. S2) also indicates a stronger role for phylogenetic signal. The 48 species represented in that analysis are generally well-studied and well-surveyed Phytophthora species, suggesting under-reporting of the distributions and host ranges of more recently described species in our full analysis may under-estimate the phylogenetic signal in these impact metrics.

**
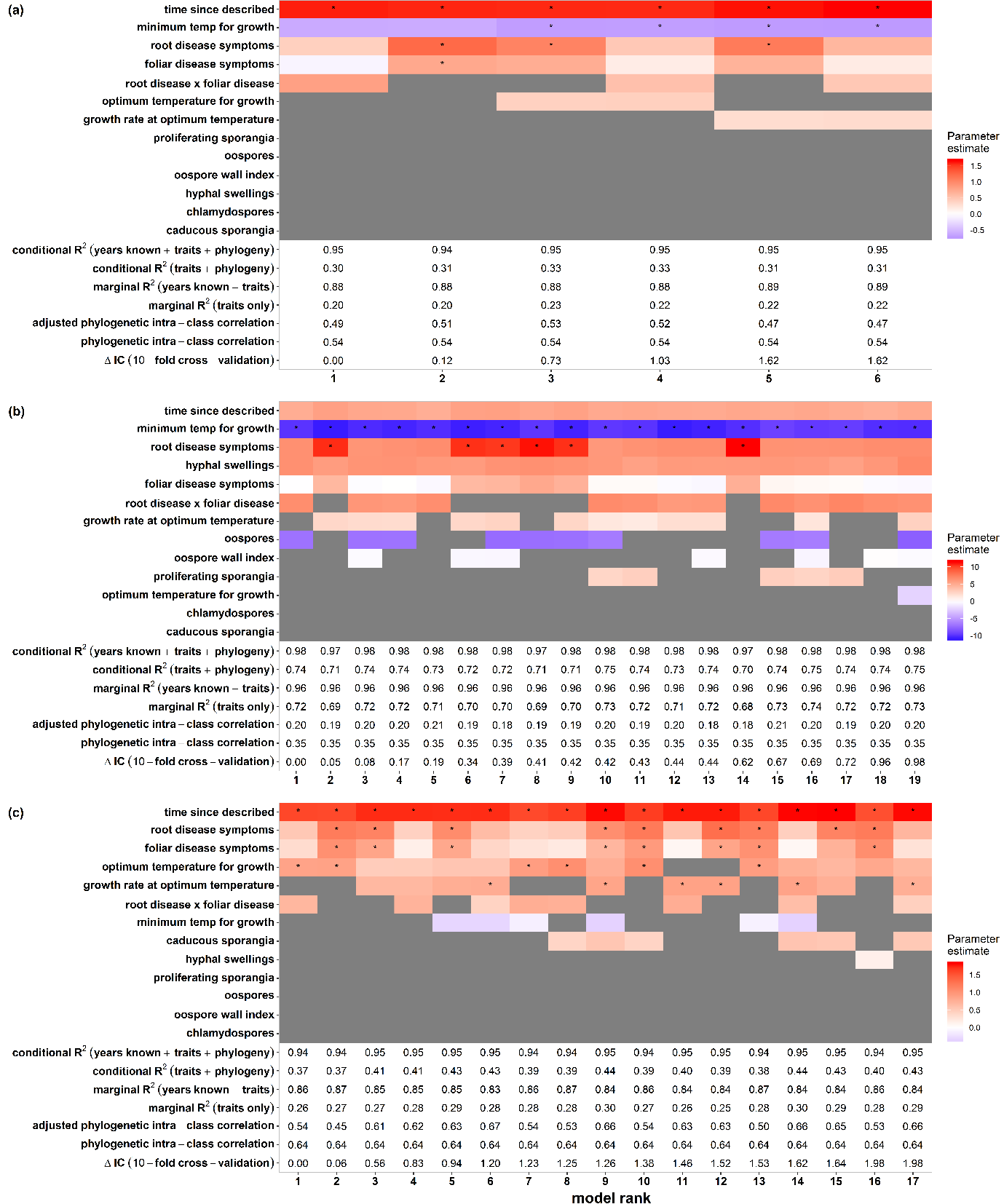
Fig. S2 Best performing trait-based models of**Phytophthora**impacts measured as (a) number of countries reached (b) absolute latitudinal limits and c) number of known host plant families using a multigene phylogeny to account for shared phylogenetic history (number of observations = 48). 2560 candidate trait-based models were ranked using a 10-fold cross-validation information criterion. The subset of best models were selected based on difference in information criterion units, *Δ*IC, of less than 2 from the best model ( >1 *ΔIC* for latitudinal limits as a large number of models (44) were within 2 *ΔIC* units). Significant effects (*) are defined as 95% credible intervals for a parameter estimate do not overlap zero. Fixed effects are ordered from top to bottom by the number of times they were present in the top model subset. Grey shading indicates predictors were absent from that model. Red and blue colours indicate positive and negative parameter estimates, respectively. Deeper colours indicate stronger effect sizes. Within-sample goodness-of-fit (*R^2^*) was quantified by partitioning variance into fixed effects (years known + traits, traits only) and phylogenetically structured error (phylogenetic intra-class correlation).**

**Appendix S4**

Over- and under-predicted *Phytophthora* species

Our models identified a number of *Phytophthora* species for which the observed number of countries reached and the number of host plant families attacked are much lower than predicted by evolutionary trait-based models. These species have similar trait values and/or are phylogenetically related to other globally widespread (Table S1) or broad host range species (Table S2). More recently described species on this list may, therefore, be those with greater potential for global impacts. For species described longer ago, taxonomic uncertainties may explain why they are under-reported. For example *P. elongata* and *P. pini* were once part of the *P. citricola* species complex and would have been recorded as *P. citricola* prior to this species complex being resolved. Others may be poorly recorded if they are opportunistic or low impact pathogens. Many species in clade 6 fall into this category (e.g. *Phytophthora humicola*).

**Table S2** The twenty *Phytophthora* species with the most under-observed global distributions (number of countries reached) compared to predictions from evolutionary trait-based models. Asterisks denote species formerly part of species complexes.

| **Species name** | **Year described** | **Phylogenetic clade** | **Observed** | **Estimate**  **[lower, upper 95% credible intervals]** | **Pearson’s residual error** |
| --- | --- | --- | --- | --- | --- |
| *Phytophthora pini* * | 1925 | 2 | 5 | 27.72 [17.63, 40.87] | -0.60 |
| *Phytophthora x heterohybrida* | 2016 | 7 | 1 | 4.14 [2.45, 7.14] | -0.57 |
| *Phytophthora humicola* | 1985 | 6 | 2 | 7.97 [4.96, 12.22] | -0.55 |
| *Phytophthora lactucae* | 2013 | 8 | 1 | 3.81 [2.14, 6.14] | -0.52 |
| *Phytophthora pistaciae* | 2001 | 7 | 1 | 3.41 [2.22, 5.11] | -0.50 |
| *Phytophthora trifolii* * | 1991 | 8 | 1 | 3.54 [2.17, 5.54] | -0.47 |
| *Phytophthora elongata* * | 2010 | 2 | 1 | 2.93 [1.85, 4.19] | -0.43 |
| *Phytophthora pisi* | 2013 | 7 | 1 | 2.89 [1.95, 4.20] | -0.43 |
| *Phytophthora psychrophila* | 2002 | 3 | 2 | 6.03 [3.47, 9.90] | -0.43 |
| *Phytophthora constricta* | 2011 | 9 | 1 | 2.95 [1.82, 4.57] | -0.42 |
| *Phytophthora crassamura* | 2015 | 6 | 1 | 2.77 [1.89, 3.92] | -0.41 |
| *Phytophthora siskiyouensis* | 2007 | 2 | 2 | 5.62 [3.12, 9.43] | -0.40 |
| *Phytophthora quercetorum* | 2008 | 4 | 1 | 2.66 [1.65, 3.87] | -0.39 |
| *Phytophthora x incrassata* | 2016 | 7 | 1 | 2.65 [1.89, 3.79] | -0.39 |
| *Phytophthora flexuosa* | 2017 | 7 | 1 | 2.40 [1.68, 3.47] | -0.38 |
| *Phytophthora amaranthi* | 2016 | 2 | 1 | 2.60 [1.50, 4.27] | -0.38 |
| *Phytophthora formosa* | 2017 | 7 | 1 | 2.44 [1.71, 3.53] | -0.37 |
| *Phytophthora intricata* | 2017 | 7 | 1 | 2.51 [1.77, 3.63] | -0.37 |
| *Phytophthora gemini* | 2011 | 6 | 1 | 2.67 [1.22, 5.86] | -0.37 |
| *Phytophthora ornamentata* | 2015 | 6 | 1 | 2.63 [1.77, 3.79] | -0.36 |

**Table S3** The twenty *Phytophthora* species with the most under-observed distributions (absolute maximum latitudinal limits) compared to predictions from evolutionary trait-based models. Asterisks denote species formerly part of species complexes.

| **Species name** | **Year described** | **Phylogenetic clade** | **Observed** | **Estimate**  **[lower, upper 95% credible intervals]** | **Pearson’s residual error** |
| --- | --- | --- | --- | --- | --- |
| *Phytophthora x heterohybrida* | 2016 | 7 | 23.75 | 47.70 [44.09, 51.10] | -1.99 |
| *Phytophthora x incrassata* | 2016 | 7 | 23.75 | 47.02 [43.56, 50.23] | -1.93 |
| *Phytophthora glovera* | 2011 | 2 | 10.79 | 35.07 [28.09, 41.88] | -1.90 |
| *Phytophthora elongata* * | 2010 | 2 | 25.73 | 48.32 [44.78, 51.48] | -1.88 |
| *Phytophthora x andina* | 2010 | 1 | 23.95 | 46.90 [41.41, 52.28] | -1.85 |
| *Phytophthora constricta* | 2011 | 9 | 25.73 | 48.09 [44.37, 51.59] | -1.84 |
| *Phytophthora flexuosa* | 2017 | 7 | 24.51 | 45.94 [42.45, 49.25] | -1.81 |
| *Phytophthora formosa* | 2017 | 7 | 24.76 | 46.15 [42.62, 49.42] | -1.76 |
| *Phytophthora intricata* | 2017 | 7 | 24.76 | 46.47 [43.05, 49.73] | -1.76 |
| *Phytophthora attenuata* | 2017 | 7 | 24.51 | 45.62 [41.99, 49.03] | -1.72 |
| *Phytophthora litoralis* | 2011 | 6 | 25.73 | 47.01 [39.72, 54.32] | -1.71 |
| *Phytophthora amaranthi* | 2016 | 2 | 23.75 | 42.53 [38.77, 46.25] | -1.55 |
| *Phytophthora versiformis* | 2017 | 11 | 25.73 | 42.57 [37.77, 47.59] | -1.38 |
| *Phytophthora frigida* | 2007 | 2 | 29.97 | 46.40 [42.73, 49.94] | -1.36 |
| *Phytophthora castaneae* | 1976 | 5 | 37.59 | 53.55 [50.03, 56.80] | -1.35 |
| *Phytophthora pistaciae* | 2001 | 7 | 32.58 | 48.18 [44.66, 51.97] | -1.30 |
| *Phytophthora gibbosa* | 2011 | 6 | 25.73 | 41.41 [37.18, 45.74] | -1.29 |
| *Phytophthora ipomoeae* | 2002 | 1 | 23.95 | 39.90 [33.70, 45.77] | -1.28 |
| *Phytophthora humicola* | 1985 | 6 | 42.80 | 57.01 [51.60, 62.44] | -1.13 |
| *Phytophthora capensis* * | 2010 | 2 | 29.00 | 42.41 [38.01, 47.04] | -1.10 |

**Table S4** The twenty *Phytophthora* species with the most under-observed host ranges (number of host plant families) compared to predictions from evolutionary trait-based models. Asterisks denote species formerly part of species complexes.

| **Species name** | **Year described** | **Phylogenetic clade** | **Observed** | **Estimate**  **[lower, upper 95% credible intervals]** | **Pearson’s residual error** |
| --- | --- | --- | --- | --- | --- |
| *Phytophthora aquimorbida* | 2012 | 9 | 0 | 3.28 [1.21, 6.64] | -0.91 |
| *Phytophthora amnicola* | 2012 | 6 | 0 | 1.50 [0.89, 2.55] | -0.75 |
| *Phytophthora fragariae* | 1940 | 7 | 1 | 6.03 [3.19, 10.02] | -0.74 |
| *Phytophthora x heterohybrida* | 2016 | 7 | 0 | 1.34 [0.82, 1.99] | -0.72 |
| *Phytophthora x incrassata* | 2016 | 7 | 0 | 1.21 [0.74, 1.80] | -0.71 |
| *Phytophthora pini* * | 1925 | 2 | 3 | 15.34 [8.27, 29.11] | -0.69 |
| *Phytophthora castaneae* | 1976 | 5 | 1 | 5.23 [2.62, 9.28] | -0.66 |
| *Phytophthora fluvialis* | 2011 | 6 | 0 | 0.96 [0.44, 2.00] | -0.64 |
| *Phytophthora mississippiae* | 2013 | 6 | 0 | 1.04 [0.45, 2.29] | -0.63 |
| *Phytophthora caryae* | 2016 | 2 | 0 | 0.79 [0.33, 1.67] | -0.57 |
| *Phytophthora riparia* | 2012 | 6 | 0 | 0.72 [0.29, 1.61] | -0.57 |
| *Phytophthora virginiana* | 2013 | 9 | 0 | 0.80 [0.29, 1.74] | -0.56 |
| *Phytophthora borealis* | 2012 | 6 | 0 | 0.71 [0.30, 1.52] | -0.56 |
| *Phytophthora hydrogena* | 2014 | 9 | 0 | 0.58 [0.20, 1.29] | -0.51 |
| *Phytophthora asparagi* | 2012 | 6 | 2 | 5.50 [2.32, 10.80] | -0.42 |
| *Phytophthora amaranthi* | 2016 | 2 | 1 | 2.46 [1.23, 4.68] | -0.38 |
| *Phytophthora ilicis* | 1957 | 3 | 1 | 2.39 [1.12, 4.77] | -0.36 |
| *Phytophthora glovera* | 2011 | 2 | 1 | 2.29 [1.14, 4.42] | -0.35 |
| *Phytophthora alticola* * | 2007 | 4 | 1 | 2.28 [1.27, 3.77] | -0.35 |
| *Phytophthora ornamentata* | 2015 | 6 | 1 | 2.04 [1.14, 3.92] | -0.33 |

Under-predicted *Phytophthora* species

Table S5 The twenty *Phytophthora* species with the most under-predicted distributions (number of countries reached) compared to predictions from evolutionary trait-based models. Asterisks denote species formerly part of species complexes.

| **Species name** | **Year described** | **Phylogenetic clade** | **Observed** | **Estimate**  **[lower, upper 95% credible intervals]** | **Pearson’s residual error** |
| --- | --- | --- | --- | --- | --- |
| *Phytophthora gregata* | 2011 | 6 | 5 | 1.24 [0.77, 1.98] | 0.78 |
| *Phytophthora plurivora* * | 2009 | 2 | 23 | 2.66 [1.78, 3.77] | 0.67 |
| *Phytophthora lacustris* | 2013 | 6 | 17 | 3.57 [2.29, 5.82] | 0.55 |
| *Phytophthora chlamydospora* | 2015 | 6 | 8 | 2.47 [1.68, 3.78] | 0.54 |
| *Phytophthora multivora* * | 2009 | 2 | 8 | 2.52 [1.70, 3.61] | 0.54 |
| *Phytophthora rubi* | 2007 | 7 | 19 | 3.74 [2.58, 5.53] | 0.53 |
| *Phytophthora quercina* | 1999 | 11 | 18 | 3.94 [2.70, 6.58] | 0.52 |
| *Phytophthora hedraiandra* | 2004 | 1 | 11 | 3.53 [1.93, 6.34] | 0.49 |
| *Phytophthora niederhauserii* | 2014 | 7 | 10 | 3.13 [1.66, 5.73] | 0.48 |
| *Phytophthora x alni* * | 2004 | 7 | 20 | 4.78 [3.35, 6.96] | 0.47 |
| *Phytophthora parvispora* | 2013 | 7 | 4 | 1.89 [1.00, 3.71] | 0.45 |
| *Phytophthora fallax* | 2006 | 9 | 5 | 2.26 [1.26, 3.92] | 0.44 |
| *Phytophthora medicaginis* * | 1991 | 8 | 15 | 4.82 [3.24, 6.81] | 0.43 |
| *Phytophthora ramorum* | 2001 | 8 | 36 | 7.15 [4.09, 12.72] | 0.43 |
| *Phytophthora intercalaris* | 2016 | 10 | 2 | 1.07 [0.63, 1.91] | 0.39 |
| *Phytophthora multivesiculata* | 1998 | 2 | 3 | 1.67 [1.00, 2.67] | 0.37 |
| *Phytophthora pinifolia* | 2008 | 6 | 2 | 1.14 [0.58, 2.33] | 0.34 |
| *Phytophthora uniformis* * | 2004 | 7 | 8 | 3.88 [2.81, 5.53] | 0.34 |
| *Phytophthora morindae* | 2010 | 10 | 2 | 1.19 [0.61, 2.49] | 0.33 |
| *Phytophthora kernoviae* | 2005 | 10 | 5 | 2.93 [1.67, 5.50] | 0.32 |

Table S6 The twenty *Phytophthora* species with the most under-predicted distributions (absolute maximum latitudinal limits) compared to predictions from evolutionary trait-based models. Asterisks denote species formerly part of species complexes.

| **Species name** | **Year described** | **Phylogenetic clade** | **Observed** | **Estimate**  **[lower, upper 95% credible intervals]** | **Pearson’s residual error** |
| --- | --- | --- | --- | --- | --- |
| *Phytophthora plurivora* * | 2009 | 2 | 68.75 | 46.45 [42.89, 50.14] | 1.82 |
| *Phytophthora gregata* | 2011 | 6 | 62.78 | 41.55 [37.27, 45.96] | 1.72 |
| *Phytophthora uniformis* * | 2004 | 7 | 68.75 | 49.96 [46.87, 52.98] | 1.58 |
| *Phytophthora rubi* | 2007 | 7 | 68.75 | 49.97 [46.11, 53.68] | 1.56 |
| *Phytophthora niederhauserii* | 2014 | 7 | 68.75 | 49.08 [42.24, 55.74] | 1.55 |
| *Phytophthora multivora* * | 2009 | 2 | 62.78 | 45.78 [42.54, 49.30] | 1.40 |
| *Phytophthora ramorum* | 2001 | 8 | 68.75 | 52.84 [48.64, 57.05] | 1.32 |
| *Phytophthora moyootj* | 2014 | 6 | 61.96 | 45.44 [37.75, 53.18] | 1.31 |
| *Phytophthora bilorbang* | 2012 | 6 | 62.78 | 46.89 [43.41, 50.96] | 1.30 |
| *Phytophthora x alni* * | 2004 | 7 | 68.75 | 52.72 [48.90, 56.31] | 1.30 |
| *Phytophthora bishii* | 2008 | 2 | 54.12 | 38.99 [33.37, 44.51] | 1.23 |
| *Phytophthora terminalis* | 2015 | 2 | 54.12 | 39.10 [34.08, 44.21] | 1.21 |
| *Phytophthora cinnamomi* | 1922 | 7 | 80.51 | 65.46 [59.34, 71.57] | 1.21 |
| *Phytophthora pisi* | 2013 | 7 | 62.78 | 48.19 [44.65, 51.89] | 1.18 |
| *Phytophthora quercina* | 1999 | 11 | 62.78 | 49.47 [46.15, 52.88] | 1.06 |
| *Phytophthora inundata* | 2003 | 6 | 68.75 | 55.90 [49.39, 62.66] | 1.05 |
| *Phytophthora fluvialis* | 2011 | 6 | 61.36 | 48.01 [40.87, 55.30] | 1.04 |
| *Phytophthora pseudosyringae* | 2003 | 3 | 62.78 | 50.51 [47.08, 54.13] | 1.03 |
| *Phytophthora occultans* | 2015 | 2 | 52.10 | 40.79 [35.65, 45.99] | 0.91 |
| *Phytophthora foliorum* | 2006 | 8 | 57.53 | 47.09 [42.48, 51.89] | 0.86 |

Table S7 The twenty Phytophthora species with the most under-predicted host ranges (number of host plant families) compared to predictions from evolutionary trait-based models. Asterisks denote species formerly part of species complexes.

| **Species name** | **Year described** | **Phylogenetic clade** | **Observed** | **Estimate**  **[lower, upper 95% credible intervals]** | **Pearson’s residual error** |
| --- | --- | --- | --- | --- | --- |
| *Phytophthora chlamydospora* | 2015 | 6 | 6 | 1.27 [0.78, 2.61] | 0.90 |
| *Phytophthora gregata* | 2011 | 6 | 4 | 1.09 [0.49, 2.33] | 0.84 |
| *Phytophthora litoralis* | 2011 | 6 | 3 | 0.88 [0.40, 1.86] | 0.84 |
| *Phytophthora lacustris* | 2013 | 6 | 4 | 1.20 [0.71, 2.43] | 0.79 |
| *Phytophthora pachypleura* * | 2014 | 2 | 18 | 2.78 [1.48, 5.99] | 0.70 |
| *Phytophthora ramorum* | 2001 | 8 | 17 | 3.08 [1.51, 8.50] | 0.64 |
| *Phytophthora kernoviae* | 2005 | 10 | 5 | 1.75 [0.79, 4.09] | 0.63 |
| *Phytophthora multivora* * | 2009 | 2 | 7 | 2.25 [1.42, 4.18] | 0.63 |
| *Phytophthora gibbosa* | 2011 | 6 | 3 | 1.22 [0.55, 2.61] | 0.61 |
| *Phytophthora tentaculata* | 1993 | 1 | 4 | 1.58 [0.90, 2.85] | 0.59 |
| *Phytophthora obscura* | 2012 | 8 | 2 | 0.91 [0.45, 1.79] | 0.52 |
| *Phytophthora attenuata* | 2017 | 7 | 2 | 1.03 [0.62, 1.59] | 0.46 |
| *Phytophthora thermophila* | 2011 | 6 | 2 | 1.00 [0.45, 2.11] | 0.45 |
| *Phytophthora siskiyouensis* | 2007 | 2 | 3 | 1.54 [0.91, 2.55] | 0.42 |
| *Phytophthora capensis* * | 2010 | 2 | 3 | 1.57 [0.67, 3.53] | 0.42 |
| *Phytophthora pseudosyringae* | 2003 | 3 | 7 | 3.38 [1.75, 6.50] | 0.42 |
| *Phytophthora parvispora* | 2013 | 7 | 5 | 2.47 [1.21, 5.80] | 0.41 |
| *Phytophthora frigida* | 2007 | 2 | 3 | 1.62 [0.92, 3.06] | 0.40 |
| *Phytophthora formosa* | 2017 | 7 | 2 | 1.14 [0.70, 1.71] | 0.39 |
| *Phytophthora plurivora* * | 2009 | 2 | 8 | 4.03 [2.29, 7.65] | 0.38 |

**References**

Bürkner, P.-C. (2017). **brms** : An *R* Package for Bayesian Multilevel Models Using *Stan*. *Journal of Statistical Software*, *80*(1), 1–28. doi: 10.18637/jss.v080.i01

Gelman, A., Hwang, J., & Vehtari, A. (2014). Understanding predictive information criteria for Bayesian models. *Statistics and Computing*, *24*(6), 997–1016. doi: 10.1007/s11222-013-9416-2

Gontcharov, A. A., Marin, B., & Melkonian, M. (2003). Are Combined Analyses Better Than Single Gene Phylogenies? A Case Study Using SSU rDNA and rbcL Sequence Comparisons in the Zygnematophyceae (Streptophyta). *Molecular Biology and Evolution*, *21*(3), 612–624. doi: 10.1093/molbev/msh052

Hadfield, J. D. (2010). MCMC Methods for Multi-Response Generalized Linear Mixed Models: The MCMCglmm R Package. *Journal of Statistical Software*, *33*, 1–22.

Harrison, X. A. (2014). Using observation-level random effects to model overdispersion in count data in ecology and evolution. *PeerJ*, *2*, e616. doi: 10.7717/peerj.616

Jung, T., Scanu, B., Brasier, C. M., Webber, J., Milenković, I., Corcobado, T., … Jung, M. H. (2020). A survey in natural forest ecosystems of vietnam reveals high diversity of both new and described phytophthora taxa including P. Ramorum. *Forests*, *11*(1), 93. doi: 10.3390/f11010093

Jung, T., Stukely, M. J. C., Hardy, G. E. S. J., White, D., Paap, T., Dunstan, W. A., & Burgess, T. I. (2011). Multiple new *Phytophthora* species from ITS clade 6 associated with natural ecosystems in Australia: Evolutionary and ecological implications. *Persoonia: Molecular Phylogeny and Evolution of Fungi*, *26*, 13–39. doi: 10.3767/003158511X557577

Martin, F. N., Blair, J. E., & Coffey, M. D. (2014). A combined mitochondrial and nuclear multilocus phylogeny of the genus *Phytophthora*. *Fungal Genetics and Biology*, *66*. doi: 10.1016/j.fgb.2014.02.006

Paradis, E., Claude, J., & Strimmer, K. (2004). Analyses of phylogenetics and evolution in R language. *Bioinformatics*, *20*, 289–290.

Piironen, J., & Vehtari, A. (2015). *Projection predictive variable selection using Stan+R*.

Wang, J., Coffey, M. D., De Maio, N., & Goss, E. M. (2020). Repeated global migrations on different plant hosts by the tropical pathogen Phytophthora palmivora. *BioRxiv*, 2020.05.13.093211. doi: 10.1101/2020.05.13.093211

Wickham, H. (2016) Hadley Wickham (2016). rvest: Easily Harvest (Scrape) Web Pages. R package version 0.3.2. https://CRAN.R-project.org/package=rvest.
